# Design, Synthesis and Biological Assessment of Rhodanine-Linked Benzenesulfonamide Derivatives as Selective and Potent Human Carbonic Anhydrase Inhibitors

**DOI:** 10.3390/molecules27228028

**Published:** 2022-11-18

**Authors:** Baijayantimala Swain, Abrar Khan, Priti Singh, Vaibhav S. Marde, Andrea Angeli, Krishna Kartheek Chinchilli, Venkata Madhavi Yaddanapudi, Simone Carradori, Claudiu T. Supuran, Mohammed Arifuddin

**Affiliations:** 1 Department of Chemical Sciences, National Institute of Pharmaceutical Education and Research (NIPER), Balanagar, Hyderabad 500037, India; 2 Neurofarba Department, University of Florence, Via Ugo Schiff 6, Sesto Fiorentino, 50019 Florence, Italy; 3 Department of Chemistry, Directorate of Distance Education, Maulana Azad National Urdu University, Gachibowli, Hyderabad 500032, India; 4 Department of Pharmacy, “G. d’Annunzio” University of Chieti-Pescara, Via dei Vestini 31, 66100 Chieti, Italy

**Keywords:** rhodanine, carbonic anhydrase, isoform selectivity, benzenesulfonamide, enzyme inhibition

## Abstract

A novel series of twenty-five rhodamine-linked benzenesulfonamide derivatives (**7a**–**u** and **9a**–**d**) were synthesized and screened for their inhibitory action against four physiologically relevant human (h) carbonic anhydrase (CA) isoforms, namely hCA I, hCA II, hCA IX, and hCA XII. All the synthesized molecules showed good to excellent inhibition against all the tested isoforms in the nanomolar range due to the presence of the sulfonamide as a zinc binding group. The target compounds were developed from indol-3-ylchalcone-linked benzenesulfonamide where the indol-3-ylchalcone moiety was replaced with rhodanine-linked aldehydes or isatins to improve the inhibition. Interestingly, the molecules were slightly more selective towards hCA IX and XII compared to hCA I and II. The most potent and efficient ones against hCA I were **7h** (*K*_I_ 22.4 nM) and **9d** (*K*_I_ 35.8 nM) compared to the standard drug AAZ (*K*_I_ 250.0 nM), whereas in case of hCA II inhibition, the derivatives containing the isatin nucleus as a tail were preferred. Collectively, all compounds were endowed with better inhibition against hCA IX compared to AAZ (*K*_I_ 25.8 nM) as well as strong potency against hCA XII. Finally, these newly synthesized molecules could be taken as potential leads for the development of isoform selective hCA IX and XII inhibitors.

## 1. Introduction

In recent years, studies of rhodanine (2-thioxothiazolidin-4-one) based molecules have been on the rise because of their wide spectrum of biological activities through different mechanisms of action, and they have become a very important group of heterocyclic compounds in drug discovery. Rhodanine consists of a five-membered ring with sulfide and amino groups at the first and third positions, respectively. The amino group could be responsible for interaction with the ligand binding site of target proteins through different types of interactions, such as hydrogen bonding as well as interactions with metal ions [1,2]. Rhodanine is mostly important in photochemistry, medicinal chemistry, biochemistry, and industry [3,4]. Several substituted rhodanine compounds have been investigated to evaluate their potential antidiabetic, anti-Alzheimer, anticancer, and antimicrobial activities [5,6]. Hence, the rhodanine structure, after different chemical modification and functionalization, could provide compounds with a broad range of biological activities [7,8,9,10].

Enzymes were usually targeted for drug discovery and development because of their vital role in many diseases. Therefore, researchers have focused on developing drug candidates that act as enzyme activators or inhibitors. Carbonic anhydrases (CAs, E.C.4.2.1.1) are metalloenzymes that play a major role in the reversible conversion of water (H_2_O) and carbon dioxide (CO_2_) into a proton (H^+^) and bicarbonate anion (HCO_3_^−^). Eight different, genetically distinct CA families are known to date: α-, β-, γ-, δ-, ζ-, θ-, η-, and ι-, depending on their catalytic activity, localization in different organisms, and sensitivity to different classes of modulators [11,12,13,14,15,16]. The α-class, which consists of 16 isoforms, is the only one present in humans. CA isoenzymes regulate many physio-pathological and biochemical processes, such as pH homeostasis, acid-base regulation, calcification, gluconeogenesis, ureagenesis, bone resorption, and tumorigenicity. Therefore, CA inhibitors (CAIs) have been experimentally and clinically proposed as anti-epileptics, diuretics, anti-glaucoma, anti-microbial, or anti-cancer agents [17,18,19,20,21,22,23,24].

**Figure 1:**
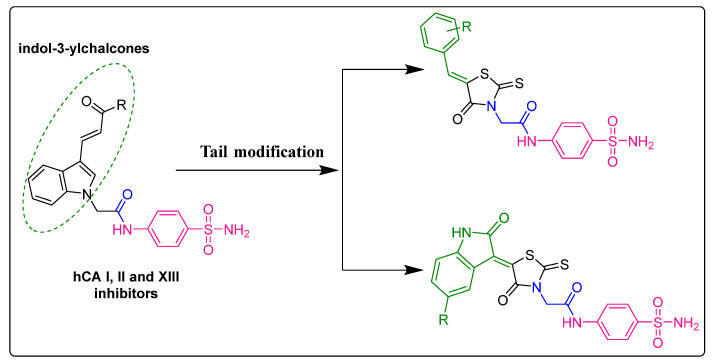
Rationale to design the target compounds.

The synthesis of rhodanine derivatives and exploring their potential inhibitory actions against various diseases are popular areas of research. Unfortunately, few efforts have been made to investigate the potential of rhodanine derivatives as hCA inhibitors [7,8,9]. Hence, in the present study, we synthesized and investigated a novel series of benzenesulfonamide-linked rhodanine derivatives for their inhibitory effects on hCA isoenzymes. Moreover, to better explore the chemical space within this privileged scaffold and to take advantage of the good inhibitory potency displayed against hCA I, II, and XIII by indole-linked benzenesulfonamide derivatives [25,26,27,28,29], we designed our derivatives functionalizing the rhodanine nucleus with both aryl and isatin rings (tail approach) to orient the isoform selectivity against four different hCA isoforms. Hence, the target compound was developed from indol-3-ylchalcone-linked benzenesulfonamide (which was previously synthesized in our lab) where the indol-3-ylchalcone moiety was replaced with rhodanine-linked aldehydes or isatins (Figure 1) in order to keep the presence of a nitrogen in this portion of the scaffold constant or not constant.

## 2. Results and Discussion

### 2.1. Chemistry

**Scheme 1:**
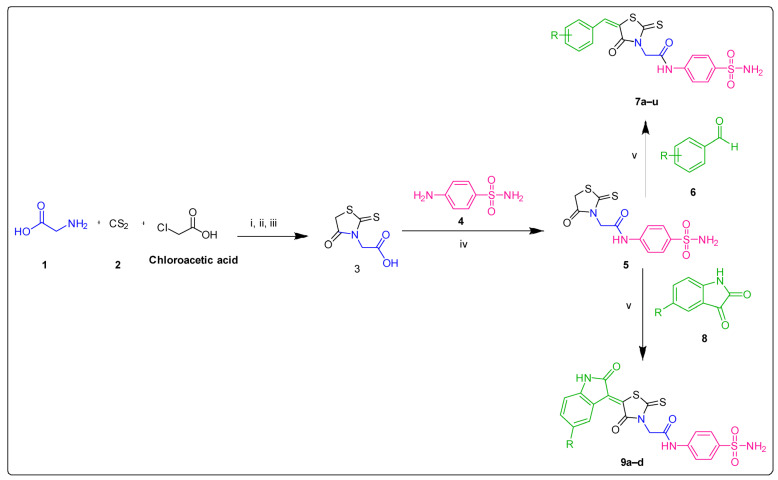
Reagent and conditions: (i) NaOH, EtOH, rt, 4–5 h; (ii) rt, 7–8 h; (iii) HCl, pH 1.0, rt, 2 h, 82%; (iv) EDCI, HOBt, DMF, rt; 7–8 h, 85%; (v) sodium acetate, acetic acid, EtOH, reflux, 4–5 h, 50–79%. For R substituents, please refer to Table 1.

The target molecules were designed using the tail approach as depicted in Scheme 1. The reactions involved the commercially available starting materials glycine (**1**) and chloroacetic acid (**2**). The intermediate 3, (2-(4-oxo-2-thioxothiazolidin-3-yl)acetic acid) was obtained via the one-pot, three component condensation of glycine, chloroacetic acid, and carbon disulfide by using a method identified in past literature [30]. The rhodamine-containing intermediate 3 (2-(4-oxo-2-thioxothiazolidin-3-yl)acetic acid) was then subjected to acid-amine coupling with sulfanilamide (**4**), according to a previously reported procedure [31], to produce intermediate **5**, which was further functionalized through Knoevenagel condensation [32] with the appropriated carbonyl compound (benzaldehydes (**6**) and isatins (**8**)) to produce the target compounds **7a**–**u** and **9a**–**d**, respectively, in good to excellent yield.

### 2.2. Carbonic Anhydrase Inhibition: Structure-Activity Relationship (SAR) Studies

**Figure 2:**
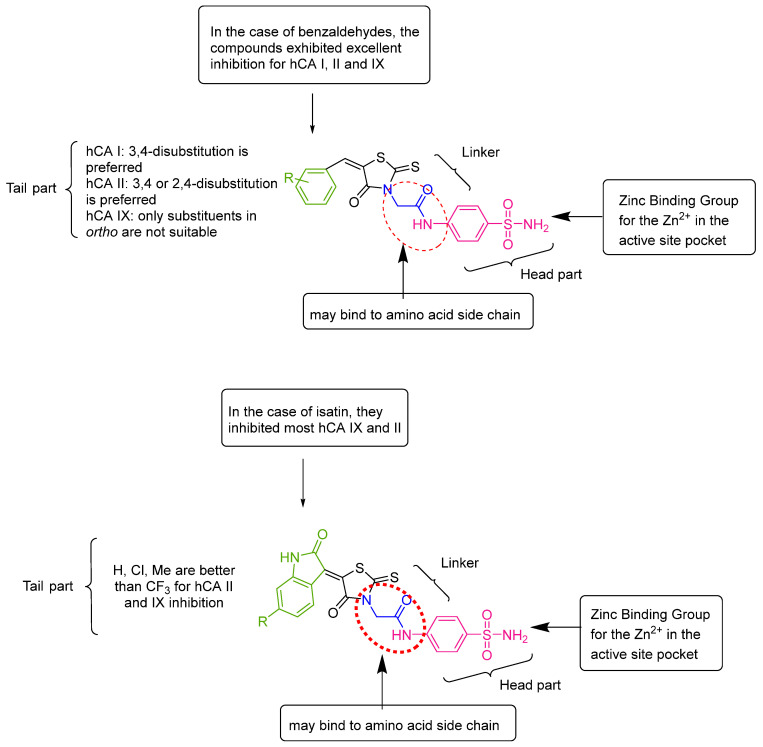
Structure–activity relationships for the target compounds against carbonic anhydrase isoforms hCA I, II, IX, and XII.

The inhibition data for the newly synthesized rhodanine-linked benzenesulfonamide derivatives were reported against two physiologically relevant cytosolic hCA I and hCA II and two tumor-associated transmembrane hCA IX and hCA XII while using acetazolamide (AAZ) as the standard drug, and the results were summarized in Table 1. All the isoenzymes belong to the human α-class of CAs. The following structure–activity relationships (SARs as reported in Figure 2) can be extrapolated as follows.

All the molecules tested against the cytosolic isoform hCA I showed good to excellent inhibition in the nanomolar range. All the compounds showed inhibition with *K*_I_ < 900 nM, except for **7c** with *K*_I_ > 1000 nM. Out of twenty-five molecules, fifteen showed excellent inhibition, with *K*_I_ inferior to the reference drug AAZ (*K*_I_ 250 nM). The most active compounds were **7h** (*K*_I_ 22.4 nM), which was 11 times more potent than AAZ, while 7k (78.6 nM), **7n** (84.0 nM), **7s** (77.9 nM), **7t** (77.5 nM), **7u** (70.6 nM), **9a** (41.6 nM), **9b** (92.2 nM), and **9d** (35.8 nM) were also more potent compared to AAZ. It was also observed that both electron-withdrawing and electron-donating substituents on the aromatic portion (R) contributed to good inhibition. Isatin-based series was slightly better than aryl-substituted rhodanines towards this isoform.

The other physiologically dominant isoform hCA II, which is also known as the anti-glaucoma drug target, was inhibited by all the tested compounds in a good to excellent nanomolar range (4.3–954.2 nM) comparable to AAZ (12.1 nM). The most active compounds were **9a** (4.3 nM, nearly three times better than AAZ), **9b** (5.5 nM), **9d** (5.2 nM), **7h** (7.3 nM), 7k (12.7 nM), **7p** (8.0 nM), **7t** (9.6 nM), and **7u** (8.9 nM). In this case, both the electron-withdrawing and electron-donating substituents on the aromatic ring (R) provided good inhibition. Compound 7c was also the least active within the series.

Excellent inhibitory activity was observed with all the synthesized compounds investigated in the low nanomolar range for the inhibition of the main tumor-associated transmembrane isozyme hCA IX. Four derivatives, namely **7b**, **7l**, **7m**, and **7o**, showed high hCA IX inhibitory activity, with *K*_I_ ranging from 28.2 to 51.5 nM, which was comparable to AAZ, whereas the remaining 21 molecules showed better inhibition (4.2–23.6 nM) than AAZ (25.8 nM). The best hCA IX inhibitors, **7g** (4.2 nM) and **9a** (4.7 nM), were nearly six times better than the standard AAZ. Collectively, the trend was flat, indicating that these rhodanine-based derivatives represent a suitable template to design potent and selective hCA IX inhibitors.

The other tumor-associated transmembrane isoenzyme hCA XII was also strongly inhibited by all the synthesized compounds, with *K*_I_ ranging from 9.0 to 258.3 nM compared to AAZ (5.7 nM). The most active compounds were **7b** (9.1 nM), **7e** (9.6 nM), **7i** (9.0 nM), **7o** (9.5 nM), **7p** (9.8 nM), **7r** (9.6 nM), **7s** (9.0 nM), and **7u** (9.5 nM). Additionally, in this study, we could not register marked variations within the series in terms of inhibitory activity, despite isatin-based compounds seemingly being slightly more active.

The molecules displayed selectivity towards hCA IX and XII compared to hCA I and II. The structure–activity relationships (Figure 2) showed that regardless of whether the molecules bared electron-donating or electron-withdrawing groups, they exhibited good inhibition. However, it was also observed that the molecules with aryl rings (**7a**–**u**) or isatins (**9a**–**d**) showed good inhibition for specific isoforms.

## 3. Materials and Methods

### 3.1. Chemistry

The target compounds were synthesized from commercially available reagents without further purification. The solvents were distilled and dried following standard methods as necessary prior to their use. All the air and moisture sensitive reactions were kept under inert conditions using clean and dried glassware and a syringe technique to transfer the solutions. The reactions were monitored via thin layer chromatography (TLC) using Merck silica gel 60F–254 plates. The synthesized molecules were purified by washing them with a mixture of 5–10% ethylacetate in hexane. The melting points were acquired using Stuart digital melting point apparatus (SMP 30) in open capillary tubes, and they were uncorrected. Nuclear Magnetic Resonance (^1^H NMR and ^13^C NMR) spectra were recorded using an Avance Bruker 500 MHz and 125 MHz spectrometer in DMSO-*d_6_* as the solvent and tetramethylsilane (TMS) as the internal standard. Chemical shifts were represented as δ values in parts per million (ppm) and coupling constants (*J*) in Hz. Multiplicities were described as s (singlet), d (doublet), t (triplet), q (quartet), m (multiplet), and dd (doublet of doublets). HRMS were determined using the Agilent QTOF mass spectrometer 6540 series instrument, and they were performed via ESI techniques at 70 eV.

### 3.2. General Procedure for the Synthesis of 2-(4-oxo-2-thioxothiazolidin-3-yl)acetic Acid (***3***)

Compound **3** was obtained from one-pot, three component condensation reaction, where glycine (2 g, 26.6 mmol) and sodium hydroxide (2.3 g, 58 mmol) were dissolved in 15 mL of ethanol followed by the dropwise addition of carbon disulfide (1.5 mL) with vigorous stirring at room temperature for 6 h. Chloroacetic acid (3 g, 31 mmol) was then added and stirring continued at room temperature for 5 h. The resultant reaction mixture was acidified with dilute HCl until pH 1.0 was achieved and stirring continued for 1 h. The progress of the reaction was checked using TLC. Twenty to thirty mL of water was then added, and the cyclized product was extracted in ethyl acetate, dried over anhydrous sodium sulfate, and evaporated under vacuum. The residue was purified by washing it with a mixture of 5–10% ethylacetate in hexane to obtain compound 3 (4.2 g, 82%).

### 3.3. General Procedure for the Synthesis of 2-(4-oxo-2-thioxothiazolidin-3-yl)-N-(4-sulfamoylphenyl)acetamide (***5***)

1-ethyl-3-(3-dimethylaminopropyl) carbodiimide hydrochloride (EDCI, 644 mg, 3.35 mmol) and 1-hydroxybenzotriazole (HOBT, 453 mg, 3.35 mmol) were added to a stirring solution of **3** (500 mg, 2.6 mmol) in *N*,*N*-dimethylformamide (DMF), which was followed by the addition of 4-aminobenzenesulfonamide (481 mg, 2.7 mmol), and the mixture was stirred vigorously at room temperature for 8 h. The progress of the reaction was checked using TLC. Forty to fifty mL of water was added to the resultant reaction mixture. The mixture was stirred vigorously to form a brownish solid precipitate, which was filtered off and dried to afford compound **5** (0.8 g, 86%).

### 3.4. General Procedure for the Synthesis of Benzylidene/Oxoindolin-Containing Thioxothiazolidin-Linked Benzenesulfonamides (***7*a**–**u**, ***9*a**–**d**)

The appropriate benzaldehyde (**6**)/isatin (**8**) (0.23 mmol), sodium acetate (37 mg, 0.45 mmol), and glacial acetic acid (0.45 mmol) were added to a stirring solution of compound 5 (80 mg, 0.23 mmol) in ethanol and then refluxed for 4–6 h. The progress of the reaction was checked using TLC, and on completion, the reaction mixture was cooled to room temperature, followed by the addition of ice-cold water, and the precipitate was filtered off, washed with water, and then dried to afford the target compounds (**7a**–**u**/**9a**–**d**) in good to excellent yield (NMR data for intermediates and final compounds are reported as Supplementary Materials).

### 3.5. Characterization Data for New Compounds

*2-(4-Oxo-2-thioxothiazolidin-3-yl)acetic acid (**3**):* light yellow solid, yield: 94%; mp: 264–268 °C; ^1^H NMR (500 MHz, DMSO-*d_6_*) δ 11.22 (s, 1H), 4.94 (s, 2H), 3.57 (s, 2H). ^13^C NMR (125 MHz, DMSO-*d_6_*) δ 193.6, 166.9, 164.2, 47.4, 44.2. HRMS (ESI-QTOF): *m*/*z* calculated for C_5_H_5_NO_3_S_2_ 191.98 [M + H]^+^ found 191.097 [M + H]^+^.

*2-(4-Oxo-2-thioxothiazolidin-3-yl)-N-(4-sulfamoylphenyl)acetamide (**5**):* light brown solid, yield: 90%; mp: 294–296 °C; ^1^ H NMR (500 MHz, DMSO-*d_6_*) δ 10.71 (s, 1H), 7.78 (d, J = 8.8 Hz, 2H), 7.69 (d, J = 8.6 Hz, 2H), 7.26 (s, 2H), 4.75 (s, 2H), 4.43 (s, 2H), 4.94 (s, 2H). ^13^C NMR (125 MHz, DMSO-*d_6_*) δ 203.6, 174.4, 164.4, 141.7, 139.3, 127.3, 119.2, 47.0, 36.6. HRMS (ESI-QTOF): *m*/*z* calculated for C_11_H_12_N_3_O_4_S_3_ 345.9990 [M + H]^+^ found 345.9997 [M + H]^+^.

*(E)-2-(5-Benzylidene-4-oxo-2-thioxothiazolidin-3-yl)-N-(4-sulfamoylphenyl)-acetamide (**7a**):* yellow solid, yield: 68%; mp: 260–262 °C; ^1^H NMR (500 MHz, DMSO-*d_6_*) δ 10.81 (s, 1H), 7.93 (s, 1H), 7.79 (d, *J* = 8.8 Hz, 2H), 7.73–7.70 (m, 4H), 7.58 (dt, *J* = 8.3, 6.8 Hz, 3H), 7.29 (s, 2H), 4.94 (s, 2H). ^13^C NMR (125 MHz, DMSO-*d_6_*) δ 194.1, 167.1, 164.3, 141.7, 139.3, 134.2, 133.3, 131.7, 131.2, 130.1, 127.3, 122.6, 119.3, 47.3. HRMS (ESI-QTOF): *m/z* calculated for C_18_H_15_N_3_NaO_4_S_3_ 456.0122 [M + Na]^+^ found 456.0144 [M + Na]^+^.

*(E)-2-(5-(4-Chlorobenzylidene)-4-oxo-2-thioxothiazolidin-3-yl)-N-(4-sulfamoylphenyl)acetamide (**7b**):* yellow solid, yield: 54%; mp: 270–272 °C; ^1^H NMR (500 MHz, DMSO-*d_6_*) δ 10.78 (s, 1H), 7.92 (s, 1H), 7.79 (d, *J* = 8.8 Hz, 2H), 7.72 (t, *J* = 8.6 Hz, 4H), 7.66 (d, *J* = 8.6 Hz, 2H), 7.26 (s, 2H), 4.94 (s, 2H). ^13^C NMR (125 MHz, DMSO-*d_6_*) δ 193.7, 167.0, 164.2, 141.7, 139.4, 136.3, 132.8, 132.2, 130.1, 127.3, 123.3, 119.3, 47.3. HRMS (ESI-QTOF): *m/z* calculated for C_18_H_14_ClN_3_O_4_S_3_ 467.9913 [M + H]^+^ found 467.1735 [M + H]^+^.

*(E)-2-(4-Oxo-2-thioxo-5-(4-(trifluoromethoxy)benzylidene)thiazolidin-3-yl)-N-(4-sulfamoylphenyl)acetamide (**7c**):* yellow solid, yield: 64%; mp: 296–298 °C; ^1^H NMR (500 MHz, DMSO-*d_6_*) δ 10.80 (s, 1H), 7.96 (s, 1H), 7.85 (d, *J* = 8.8 Hz, 2H), 7.79 (d, *J* = 8.8 Hz, 2H), 7.72 (d, *J* = 8.8 Hz, 2H), 7.58 (d, *J* = 8.3 Hz, 2H), 7.28 (s, 2H), 4.95 (s, 2H). ^13^C NMR (125 MHz, DMSO-*d_6_*) δ 193.8, 167.0, 164.2, 150.1, 141.7, 139.4, 133.3, 132.5, 127.3, 123.7, 122.2, 119.3, 47.3. HRMS (ESI-QTOF): *m/z* calculated for C_19_H_14_F_3_N_3_NaO_5_S_3_ 539.9945 [M + Na]^+^ found 539.998 [M + Na]^+^.

*(E)-2-(5-(2-Fluorobenzylidene)-4-oxo-2-thioxothiazolidin-3-yl)-N-(4-sulfamoylphenyl)acetamide (**7d**):* yellow solid, yield: 55%; mp: 230–232 °C; ^1^H NMR (500 MHz, DMSO-*d_6_*) δ 10.83 (s, 1H), 7.87 (s, 1H), 7.79 (d, *J* = 8.8 Hz, 2H), 7.72 (d, *J* = 8.9 Hz, 2H), 7.66 (d, *J* = 7.5 Hz, 1H), 7.63 (d, *J* = 7.7 Hz, 1H), 7.45 (t, *J* = 8.0 Hz, 2H), 7.30 (s, 2H), 4.95 (s, 2H). ^13^C NMR (125 MHz, DMSO-*d_6_*) δ 193.5, 166.8, 164.2, 148.9, 141.6, 139.4, 136.3, 134.9, 131.7, 131.6, 127.3, 125.7, 125.5, 119.3, 47.4. HRMS (ESI-QTOF): *m/z* calculated for C_18_H_14_FN_3_NaO_4_S_3_ 474.0028 [M + Na]^+^ found 474.0048 [M + Na]^+^.

*(E)-2-(4-Oxo-2-thioxo-5-(4-(trifluoromethyl)benzylidene)thiazolidin-3-yl)-N-(4-sulfamoylphenyl)acetamide (**7e**):* yellow solid, yield: 60%; mp: 291–293 °C; ^1^H NMR (500 MHz, DMSO-*d_6_*) δ 10.82 (s, 1H), 8.01 (s, 1H), 7.93 (q, *J* = 8.7 Hz, 4H), 7.79 (d, *J* = 8.7 Hz, 2H), 7.72 (d, *J* = 8.7 Hz, 2H), 7.29 (s, 2H), 4.95 (s, 2H). ^13^C NMR (125 MHz, DMSO-*d_6_*) δ 193.7, 166.9, 164.2, 141.6, 139.4, 137.2, 132.2, 131.6, 130.8, 130.5, 127.3, 126.8, 126.7, 125.6, 125.3, 123.2, 119.3, 47.3. HRMS (ESI-QTOF): *m/z* calculated for C_19_H_14_F_3_N_3_NaO_4_S_3_ 523.9996 [M + Na]^+^ found 523.9992 [M + Na]^+^.

*(E)-2-(5-(4-(Allyloxy)benzylidene)-4-oxo-2-thioxothiazolidin-3-yl)-N-(4-sulfamoylphenyl)acetamide (**7f**):* yellow solid, yield: 58%; mp: 262–264 °C; ^1^H NMR (500 MHz, DMSO-*d_6_*) δ 10.80 (s, 1H), 7.88 (s, 1H), 7.79 (d, *J* = 8.8 Hz, 2H), 7.72 (d, *J* = 8.9 Hz, 2H), 7.67 (d, *J* = 8.9 Hz, 2H), 7.29 (s, 2H), 7.17 (d, *J* = 8.9 Hz, 2H), 6.07 (ddt, *J* = 17.2, 10.5, 5.3 Hz, 1H), 5.46–5.41 (m, 1H), 5.31 (dd, *J* = 10.5, 1.5 Hz, 1H), 4.93 (s, 2H), 4.71–4.69 (m, 2H). ^13^C NMR (125 MHz, DMSO-*d_6_*) δ 193.8, 167.1, 164.3, 161.1, 141.7, 139.3, 134.4, 133.5, 127.3, 126.0, 119.3, 118.5, 116.3, 69.0, 47.2. HRMS (ESI-QTOF): *m/z* calculated for C_21_H_19_N_3_NaO_5_S_3_ 512.0385 [M + Na]^+^ found 512.0371 [M + Na]^+^.

*(E)-2-(4-Oxo-2-thioxo-5-(3,4,5-trimethoxybenzylidene)thiazolidin-3-yl)-N-(4-sulfamoylphenyl)acetamide (**7g**):* yellow solid, yield: 56%; mp: 290–292 °C; ^1^H NMR (500 MHz, DMSO-*d_6_*) δ 10.81 (s, 1H), 7.87 (s, 1H), 7.79 (d, *J* = 8.9 Hz, 2H), 7.72 (d, *J* = 8.9 Hz, 2H), 7.29 (s, 2H), 7.02 (s, 2H), 4.95 (s, 2H), 3.88 (s, 6H), 3.77 (s, 3H). ^13^C NMR (125 MHz, DMSO-*d_6_*) δ 193.8, 167.0, 164.3, 153.8, 141.7, 140.6, 139.3, 134.6, 128.8, 127.3, 121.4, 119.2, 108.7, 60.7, 56.6, 47.3. HRMS (ESI-QTOF): *m/z* calculated for C_21_H_21_N_3_NaO_7_S_3_ 546.0439 [M + Na]^+^ found 546.8308 [M + Na]^+^.

*(E)-2-(5-(3-Ethoxy-4-hydroxybenzylidene)-4-oxo-2-thioxothiazolidin-3-yl)-N-(4-sulfamoylphenyl)acetamide (**7h**):* yellow solid, yield: 52%; mp: 278–280 °C; ^1^H NMR (500 MHz, DMSO-*d_6_*) δ 10.80 (s, 1H), 10.16 (s, 1H), 7.82 (s, 1H), 7.79 (d, *J* = 8.8 Hz, 2H), 7.71 (d, *J* = 8.8 Hz, 2H), 7.29 (s, 2H), 7.24 (d, *J* = 1.7 Hz, 1H), 7.19 (dd, *J* = 8.4, 1.8 Hz, 1H), 7.00 (d, *J* = 8.3 Hz, 1H), 4.93 (s, 2H), 4.12 (q, *J* = 6.9 Hz, 2H), 1.39 (t, *J* = 6.9 Hz, 3H). ^13^C NMR (125 MHz, DMSO-*d_6_*) δ 193.8, 167.1, 164.3, 151.2, 147.8, 141.7, 139.3, 135.3, 127.3, 125.9, 124.8, 119.3, 118.0, 117.0, 116.2, 64.4, 47.2, 15.1. HRMS (ESI-QTOF): *m/z* calculated for C_20_H_19_N_3_NaO_6_S_3_ 516.0334 [M + Na]^+^ found 516.0321 [M + Na]^+^.

*(E)-2-(5-(4-Cyanobenzylidene)-4-oxo-2-thioxothiazolidin-3-yl)-N-(4-sulfamoylphenyl)acetamide (**7i**):* yellow solid, yield: 56%; mp: 308–310 °C; ^1^H NMR (500 MHz, DMSO-*d_6_*) δ 10.82 (s, 1H), 8.03 (d, *J* = 7.9 Hz, 2H), 7.98 (s, 1H), 7.88 (d, *J* = 7.9 Hz, 2H), 7.79 (d, *J* = 8.3 Hz, 2H), 7.72 (d, *J* = 8.3 Hz, 2H), 7.30 (s, 2H), 4.95 (s, 2H). ^13^C NMR (125 MHz, DMSO-*d_6_*) δ 198.3, 171.6, 168.9, 146.4, 144.1, 142.4, 138.4, 136.6, 136.3, 132.1, 130.9, 123.9, 123.5, 117.8, 52.1. HRMS (ESI-QTOF): *m/z* calculated for C_19_H_14_N_4_NaO_4_S_3_ 481.0075 [M + Na]^+^ found 481.0072 [M + Na]^+.^.

*(E)-2-(5-(3-Methoxybenzylidene)-4-oxo-2-thioxothiazolidin-3-yl)-N-(4-sulfamoylphenyl)acetamide (**7j**):* yellow solid, yield: 52%; mp: 258–260 °C; ^1^H NMR (500 MHz, DMSO-*d_6_*) δ 10.80 (s, 1H), 7.90 (s, 1H), 7.79 (d, *J* = 8.8 Hz, 2H), 7.72 (d, *J* = 8.9 Hz, 2H), 7.51 (t, *J* = 8.2 Hz, 1H), 7.29 (s, 2H), 7.26 (dd, *J* = 4.3, 2.3 Hz, 2H), 7.14 (dd, *J* = 8.3, 1.8 Hz, 1H), 4.94 (s, 2H), 3.84 (s, 3H). ^13^C NMR (125 MHz, DMSO-*d_6_*) δ 194.0, 167.0, 164.2, 160.2, 141.7, 139.3, 134.7, 134.2, 131.2, 127.3, 123.0, 122.9, 119.3, 117.7, 116.4, 55.8, 47.3. HRMS (ESI-QTOF): *m/z* calculated for C_19_H_17_N_3_NaO_5_S_3_ 486.0219 [M + Na]^+^ found 486.0228 [M + Na]^+^.

*(E)-2-(5-(4-Methoxybenzylidene)-4-oxo-2-thioxothiazolidin-3-yl)-N-(4-sulfamoylphenyl)acetamide (**7k**):* yellow solid, yield: 55%; mp: 275–277 °C; ^1^H NMR (500 MHz, DMSO-*d_6_*) δ 10.81 (s, 1H), 7.88 (s, 1H), 7.79 (d, *J* = 8.4 Hz, 2H), 7.72 (d, *J* = 8.5 Hz, 2H), 7.68 (d, *J* = 8.4 Hz, 2H), 7.29 (s, 2H), 7.16 (d, *J* = 8.4 Hz, 2H), 4.94 (s, 2H), 3.87 (s, 3H). ^13^C NMR (125 MHz, DMSO-*d_6_*) δ 193.9, 167.2, 164.3, 162.2, 141.7, 139.3, 134.4, 133.6, 127.3, 125.9, 119.3, 119.2, 115.7, 115.0, 56.1, 47.3. HRMS (ESI-QTOF): *m/z* calculated for C_19_H_17_N_3_NaO_5_S_3_ 486.0219 [M + Na]^+^ found 486.0222 [M + Na]^+^.

*(E)-2-(5-(4-Isopropylbenzylidene)-4-oxo-2-thioxothiazolidin-3-yl)-N-(4-sulfamoylphenyl)acetamide (**7l**):* yellow solid, yield: 56%; mp: 268–270 °C; ^1^H NMR (500 MHz, DMSO-*d_6_*) δ 10.80 (s, 1H), 7.89 (s, 1H), 7.79 (d, *J* = 8.9 Hz, 2H), 7.71 (d, *J* = 8.9 Hz, 2H), 7.64 (d, *J* = 8.3 Hz, 2H), 7.48 (d, *J* = 8.3 Hz, 2H), 7.29 (s, 2H), 4.94 (s, 2H), 2.98 (dt, *J* = 13.7, 6.9 Hz, 1H), 1.24 (d, *J* = 6.9 Hz, 6H). ^13^C NMR (125 MHz, DMSO-*d_6_*) δ 194.0, 167.1, 164.3, 152.8, 141.7, 139.3, 134.3, 131.5, 131.1, 128.1, 127.3, 121.4, 119.3, 47.2, 33.9, 23.9. HRMS (ESI-QTOF): *m/z* calculated for C_21_H_21_N_3_NaO_4_S_3_ 498.0592 [M + Na]^+^ found 498.0626 [M + Na]^+^.

*(E)-2-(4-Oxo-5-(3-phenoxybenzylidene)-2-thioxothiazolidin-3-yl)-N-(4-sulfamoylphenyl)acetamide (**7m**):* yellow solid, yield: 52%; mp: 287–289 °C; ^1^H NMR (500 MHz, DMSO-*d_6_*) δ 10.79 (s, 1H), 7.91 (s, 1H), 7.79 (d, *J* = 8.8 Hz, 2H), 7.71 (d, *J* = 8.8 Hz, 2H), 7.60 (t, *J* = 8.0 Hz, 1H), 7.46 (t, *J* = 8.0 Hz, 3H), 7.29 (s, 3H), 7.23 (t, *J* = 7.4 Hz, 1H), 7.19 (dd, *J* = 8.2, 2.0 Hz, 1H), 7.12 (d, *J* = 7.8 Hz, 2H), 4.93 (s, 2H). ^13^C NMR (125 MHz, DMSO-*d_6_*) δ 193.8, 166.9, 164.2, 158.1, 156.2, 141.7, 139.3, 135.2, 133.5, 131.7, 130.7, 127.3, 125.7, 124.7, 123.5, 121.3, 120.2, 119.7, 119.3, 47.3. HRMS (ESI-QTOF): *m/z* calculated for C_24_H_19_N_3_NaO_5_S_3_ 548.0385 [M + Na]^+^ found 548.0387 [M + Na]^+^.

*(E)-2-(5-(3,4-Dimethoxybenzylidene)-4-oxo-2-thioxothiazolidin-3-yl)-N-(4-sulfamoylphenyl)acetamide (**7n**):* yellow solid, yield: 63%; mp: 288–290 °C; ^1^H NMR (500 MHz, DMSO-*d_6_*) δ 10.79 (s, 1H), 7.87 (s, 1H), 7.79 (d, *J* = 8.8 Hz, 2H), 7.72 (d, *J* = 8.8 Hz, 2H), 7.32 (d, *J* = 1.9 Hz, 1H), 7.28 (s, 3H), 7.19 (d, *J* = 8.5 Hz, 1H), 4.94 (s, 2H), 3.86 (s, 6H). ^13^C NMR (125 MHz, DMSO-*d_6_*) δ 193.8, 167.1, 164.3, 152.1, 149.6, 141.7, 139.3, 134.8, 127.3, 126.1, 125.5, 119.4, 119.3, 114.3, 112.8, 56.3, 56.1, 47.3. HRMS (ESI-QTOF): *m/z* calculated for C_20_H_19_N_3_NaO_6_S_3_ 516.0334 [M + Na]^+^ found 516.0312 [M + Na]^+^.

*(E)-2-(4-Oxo-2-thioxo-5-(2,4,5-trimethoxybenzylidene)thiazolidin-3-yl)-N-(4-sulfamoylphenyl)acetamide (**7o**):* yellow solid, yield: 59%; mp: 288–290 °C; ^1^H NMR (500 MHz, DMSO-*d_6_*) δ 10.79 (s, 1H), 7.97 (s, 1H), 7.79 (d, *J* = 8.8 Hz, 2H), 7.71 (d, *J* = 8.8 Hz, 2H), 7.28 (s, 2H), 6.99 (s, 1H), 6.84 (s, 1H), 4.92 (s, 2H), 3.96 (s, 3H), 3.93 (s, 3H), 3.81 (s, 3H). ^13^C NMR (125 MHz, DMSO-*d_6_*) δ 194.2, 167.3, 164.4, 155.3, 154.4, 143.7, 141.7, 139.3, 130.2, 127.3, 119.3, 118.5, 113.2, 112.9, 98.1, 56.9, 56.6, 56.5, 47.2. HRMS (ESI-QTOF): *m/z* calculated for C_21_H_21_N_3_NaO_7_S_3_ 546.0439 [M + Na]^+^ found 546.0451 [M + Na]^+^.

*(E)-2-(5-(2,4-Dimethoxybenzylidene)-4-oxo-2-thioxothiazolidin-3-yl)-N-(4-sulfamoylphenyl)acetamide (**7p**):* yellow solid, yield: 60%; mp: 275–277 °C; ^1^H NMR (500 MHz, DMSO-*d_6_*) δ 10.78 (s, 1H), 7.96 (s, 1H), 7.79 (d, *J* = 8.8 Hz, 2H), 7.71 (d, *J* = 8.9 Hz, 2H), 7.47 (d, *J* = 8.7 Hz, 1H), 7.28 (s, 2H), 6.76 (dd, *J* = 8.6, 2.3 Hz, 1H), 6.74 (d, *J* = 2.3 Hz, 1H), 4.92 (s, 2H), 3.95 (s, 3H), 3.89 (s, 3H). ^13^C NMR (125 MHz, DMSO-*d_6_*) δ 194.4, 167.3, 164.6, 164.4, 160.6, 141.7, 139.3, 132.7, 129.8, 127.3, 119.3, 118.8, 114.7, 107.7, 99.2, 56.5, 56.3, 47.2. HRMS (ESI-QTOF): *m/z* calculated for C_20_H_19_N_3_NaO_5_S_3_ 500.0385 [M + Na]^+^ found 500.0369 [M + Na]^+^.

*(E)-2-(4-Oxo-2-thioxo-5-(2,4,6-trimethoxybenzylidene)thiazolidin-3-yl)-N-(4-sulfamoylphenyl)acetamide (**7q**):* orange solid, yield: 56%; mp: 273–275 °C; ^1^H NMR (500 MHz, DMSO-*d_6_*) δ 10.77 (s, 1H), 8.04 (s, 1H), 7.78 (d, *J* = 8.8 Hz, 2H), 7.71 (d, *J* = 8.8 Hz, 2H), 7.28 (s, 2H), 6.37 (s, 2H), 4.90 (s, 2H), 3.93 (s, 6H), 3.90 (s, 3H). ^13^C NMR (125 MHz, DMSO-*d_6_*) δ 195.7, 167.8, 165.9, 164.5, 160.6, 141.8, 139.3, 128.1, 127.3, 119.5, 119.2, 104.0, 91.7, 56.4, 56.4, 47.2. HRMS (ESI-QTOF): *m/z* calculated for C_21_H_21_N_3_NaO_7_S_3_ 546.0439 [M + Na]^+^ found 546.0428 [M + Na]^+^.

*(E)-2-(5-(3,4-Difluorobenzylidene)-4-oxo-2-thioxothiazolidin-3-yl)-N-(4-sulfamoylphenyl)acetamide (**7r**):* yellow solid, yield: 52%; mp: 274–276 °C; ^1^H NMR (500 MHz, DMSO-*d_6_*) δ 10.82 (s, 1H), 7.91 (s, 1H), 7.87–7.82 (m, 1H), 7.79 (d, *J* = 8.8 Hz, 2H), 7.71 (d, *J* = 8.8 Hz, 2H), 7.69–7.64 (m, 1H), 7.57 (s, 1H), 7.30 (s, 2H), 4.94 (s, 2H). ^13^C NMR (125 MHz, DMSO-*d_6_*) δ 198.4, 171.7, 169.0, 157.0, 156.9, 156.0, 155.9, 155.0, 154.0, 153.9, 146.4, 144.1, 136.7, 135.9, 132.9, 132.9, 132.8, 132.1, 132.0, 128.7, 125.3, 125.2, 124.1, 124.0, 52.1. HRMS (ESI-QTOF): *m/z* calculated for C_18_H_13_F_2_N_3_NaO_4_S_3_ 491.9934 [M + Na]^+^ found 491.9921 [M + Na]^+^.

*(E)-2-(5-(3-Nitrobenzylidene)-4-oxo-2-thioxothiazolidin-3-yl)-N-(4-sulfamoylphenyl)acetamide (**7s**):* orange solid, yield: 55%; mp: 295–297 °C; ^1^H NMR (500 MHz, DMSO-*d_6_*) δ 10.81 (s, 1H), 8.57 (s, 1H), 8.36 (d, *J* = 11.2 Hz, 1H), 8.11 (d, *J* = 7.4 Hz, 2H), 7.88 (t, *J* = 8.0 Hz, 1H), 7.79 (d, *J* = 8.9 Hz, 2H), 7.72 (d, *J* = 8.9 Hz, 2H), 7.28 (s, 2H), 4.96 (s, 2H). ^13^C NMR (125 MHz, DMSO-*d_6_*) δ 193.4, 166.8, 164.2, 148.9, 141.6, 139.4, 136.3, 134.9, 131.7, 131.6, 127.3, 125.7, 125.5, 119.3, 47.4. HRMS (ESI-QTOF): *m/z* calculated for C_18_H_14_N_4_NaO_6_S_3_ 500.9973 [M + Na]^+^ found500.9985 [M + Na]^+^.

*(E)-2-(5-(4-Hydroxy-3,5-dimethoxybenzylidene)-4-oxo-2-thioxothiazolidin-3-yl)-N-(4-sulfamoylphenyl)acetamide (**7t**):* orange solid, yield: 59%; mp: 291–293 °C; ^1^H NMR (500 MHz, DMSO-*d_6_*) δ 10.81 (s, 1H), 9.63 (s, 1H), 7.83 (s, 1H), 7.79 (d, *J* = 8.8 Hz, 2H), 7.72 (d, *J* = 8.8 Hz, 2H), 7.29 (s, 2H), 6.99 (s, 2H), 4.94 (s, 2H), 3.87 (s, 6H). ^13^C NMR (125 MHz, DMSO-*d_6_*) δ 193.7, 167.1, 164.3, 148.9, 141.7, 140.2, 139.3, 135.5, 127.3, 123.7, 119.3, 118.4, 109.3, 56.6, 49.1. HRMS (ESI-QTOF): *m/z* calculated for C_20_H_19_N_3_NaO_7_S_3_ 532.0283 [M + Na]^+^ found 532.0274 [M + Na]^+^.

*(E)-2-(5-(4-Nitrobenzylidene)-4-oxo-2-thioxothiazolidin-3-yl)-N-(4-sulfamoylphenyl)acetamide (**7u**):* yellow solid, yield: 57%; mp: 258–260 °C; ^1^H NMR (500 MHz, DMSO-*d_6_*) δ 10.86 (s, 1H), 8.39 (d, *J* = 8.7 Hz, 2H), 8.04 (s, 1H), 7.97 (d, *J* = 8.8 Hz, 2H), 7.79 (d, *J* = 8.8 Hz, 2H), 7.72 (d, *J* = 8.8 Hz, 2H), 7.30 (s, 2H), 4.96 (s, 2H). ^13^C NMR (125 MHz, DMSO-*d_6_*) δ 193.6, 166.9, 164.2, 148.3, 141.6, 139.4, 139.4, 132.1, 131.3, 127.3, 126.8, 124.9, 119.3, 47.4. HRMS (ESI-QTOF): *m/z* calculated for C_18_H_14_N_4_NaO_6_S_3_ 500.9973 [M + Na]^+^ found 500.9985 [M + Na]^+^.

*(Z)-2-(4-Oxo-5-(2-oxoindolin-3-ylidene)-2-thioxothiazolidin-3-yl)-N-(4-sulfamoylphenyl)acetamide (**9a**):* orange solid, yield: 76%; mp: 230–233 °C; ^1^H NMR (500 MHz, DMSO-*d_6_*) δ 11.34 (s, 1H), 10.82 (s, 1H), 8.79 (d, *J* = 7.9 Hz, 1H), 7.79 (d, *J* = 8.8 Hz, 2H), 7.74–7.71 (m, 2H), 7.45 (dd, *J* = 11.1, 4.3 Hz, 1H), 7.29 (s, 2H), 7.10 (t, *J* = 7.8 Hz, 1H), 6.99 (d, *J* = 7.8 Hz, 1H), 4.99 (s, 2H). ^13^C NMR (125 MHz, DMSO-*d_6_*) δ 198.2, 168.4, 166.9, 164.3, 145.5, 141.7, 139.4, 134.0, 130.3, 128.4, 127.3, 126.7, 122.8, 120.2, 119.4, 111.4, 47.0. HRMS (ESI-QTOF): *m/z* calculated for C_19_H_14_N_4_NaO_5_S_3_ 497.0024 [M + Na]^+^ found 497.0019 [M + Na]^+^.

*(Z)-2-(5-(5-Methyl-2-oxoindolin-3-ylidene)-4-oxo-2-thioxothiazolidin-3-yl)-N-(4-sulfamoylphenyl)acetamide (**9b**):* orange solid, yield: 79%; mp: 259–261 °C; ^1^H NMR (500 MHz, DMSO-*d_6_*) δ 11.13 (s, 1H), 10.81 (s, 1H), 8.45 (t, *J* = 5.2 Hz, 1H), 7.81–7.77 (m, 2H), 7.73–7.70 (m, 2H), 7.28 (s, 2H), 7.07 (dd, *J* = 8.6, 2.7 Hz, 1H), 6.90 (d, *J* = 8.5 Hz, 1H), 4.99 (s, 2H), 3.77 (s, 3H). ^13^C NMR (125 MHz, DMSO-*d_6_*) δ 198.3, 168.4, 167.0, 164.3, 155.2, 141.6, 139.4, 139.4, 130.5, 127.3, 127.1, 120.8, 120.0, 119.4, 113.7, 111.9, 56.1, 47.0. HRMS (ESI-QTOF): *m/z* calculated for C_20_H_16_N_4_NaO_5_S_3_ 511.0181 [M + Na]^+^ found 511.0201 [M + Na]^+^.

*(Z)-2-(4-Oxo-5-(2-oxo-5-(trifluoromethyl)indolin-3-ylidene)-2-thioxothiazolidin-3-yl)-N-(4-sulfamoylphenyl)acetamide (**9c**):* brown solid, yield: 73%; mp: 246–248 °C; ^1^H NMR (500 MHz, DMSO-*d_6_*) δ 11.53 (s, 1H), 10.82 (s, 1H), 8.78 (s, 1H), 7.79 (d, *J* = 8.8 Hz, 2H), 7.72 (d, *J* = 8.8 Hz, 2H), 7.49 (d, *J* = 8.4 Hz, 1H), 7.28 (s, 2H), 7.09 (d, *J* = 8.6 Hz, 1H), 5.00 (s, 2H). ^13^C NMR (125 MHz, DMSO-*d_6_*) δ 197.8, 168.4, 167.2, 164.2, 144.4, 141.6, 139.4, 132.7, 127.3, 126.7, 125.4, 121.0, 119.4, 112.4, 47.1. HRMS (ESI-QTOF): *m/z* calculated for C_20_H_13_F_3_N_4_NaO_5_S_3_ 564.9898 [M + Na]^+^ found 564.9867 [M + Na]^+^.

*(Z)-2-(5-(5-Chloro-2-oxoindolin-3-ylidene)-4-oxo-2-thioxothiazolidin-3-yl)-N-(4-sulfamoylphenyl)acetamide (**9d**):* orange solid, yield: 78%; mp: 251–253 °C; ^1^H NMR (500 MHz, DMSO-*d_6_*) δ 11.49 (s, 1H), 10.84 (s, 1H), 8.81 (d, *J* = 2.1 Hz, 1H), 7.79 (d, *J* = 8.8 Hz, 2H), 7.72 (d, *J* = 8.8 Hz, 2H), 7.52 (dd, *J* = 8.4, 2.2 Hz, 1H), 7.29 (s, 2H), 7.02 (d, *J* = 8.4 Hz, 1H), 5.00 (s, 2H). ^13^C NMR (125 MHz, DMSO-*d_6_*) δ 197.8, 168.2, 167.1, 164.2, 144.1, 141.6, 139.4, 133.1, 132.2, 127.5, 127.3, 126.6, 125.4, 121.5, 119.4, 112.8, 47.0. HRMS (ESI-QTOF): *m/z* calculated for C_19_H_13_ClN_4_NaO_5_S_3_ 530.9634 [M + Na]^+^ found 530.9647 [M + Na]^+^.

### 3.6. Carbonic Anhydrase Inhibition Assay

An SX.18MV-R Applied Photophysics (Oxford, UK) stopped-flow instrument was used to assay the inhibition of various CA isozymes [33]. Phenol Red (at a concentration of 0.2 mM) was used as an indicator, working at an absorbance maximum of 557 nm with 10 mM Hepes (pH 7.4) as a buffer and 10 mM NaClO_4_ to maintain constant ionic strength (this anion was not inhibitory in the used concentration), following the CA-catalyzed CO_2_ hydration reaction for a period of 5–10 s. Saturated CO_2_ solutions in water at 25 °C were used as substrate. Stock solutions of inhibitors were prepared at a concentration of 10 mM (in DMSO-water 1:1, *v*/*v*) with dilutions up to 0.01 nM done with the assay buffer mentioned above. At least seven different inhibitor concentrations were used to measure the inhibition constant. Inhibitor and enzyme solutions were preincubated together for 10 min at room temperature prior to assay to allow for the formation of the E-I complex. Triplicate experiments were done for each inhibitor concentration, and the values reported throughout the paper were the means of these results. The inhibition constants were obtained via non-linear, least-squares methods using the Cheng–Prusoff equation, as reported earlier [34], and they represented the means of at least three different determinations. All CA isozymes used in this study were recombinant proteins obtained by our group, as reported earlier, and their concentrations in the assay system were 5–12 nM [35,36,37].

## 4. Conclusions

The present research work explained the design and synthesis of novel benzenesulfonamide-linked rhodanine derivatives (**7a**–**u**, **9a**–**d**) as crucial and selective human carbonic anhydrase inhibitors. All the tested hCA isoforms (I, II, IX, XII) were affected by the newly synthesized molecules in variable degrees of inhibition. All the molecules showed excellent inhibition in the nanomolar range. However, the selectivity was more pronounced towards hCA IX and XII. The most potent and efficient compounds against hCA I were **7h** (22.4 nM) and **9d** (35.8 nM) compared to standard AAZ (250.0 nM), whereas in the case of hCA II, the isatin derivatives **9a** (4.3 nM), **9b** (5.5 nM), and **9d** (5.2 nM) exhibited excellent potency compared to standard AAZ (12.1 nM). The compounds which showed the best inhibition against isoform hCA IX were **7g** (4.2 nM) and **9a** (4.7 nM) compared to standard AAZ (25.8 nM). Conversely, for hCA XII, all the compounds exhibited moderate potencies, and the best among them were **7i** (9.0 nM) and **7s** (9.0 nM) compared to standard AAZ (5.7 nM). It was also noticed that both aryls and isatins with electron-donating and electron-withdrawing groups showed good inhibition potencies. From the results obtained in the present study, it is concluded that these newly synthesized benzenesulfonamide-linked rhodanine derivatives are very interesting lead molecules for the design and synthesis of isoform selective inhibitors of tumor-associated isoforms.

## Figures and Tables

**Table 1:** Inhibition of hCA isoforms I, II, IX, and XII with *K*_I_ (nM) data for compounds **7a**–**u** and **9a**–**d** and acetazolamide (AAZ) as the standard inhibitor by means of the stopped-flow carbon dioxide assays.

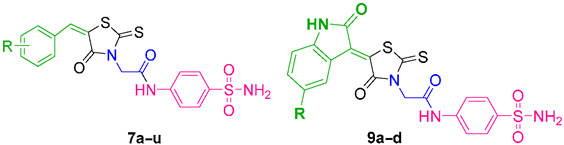					
*K*_I_ (nM) *					
**Compound**	**R**	**hCA I**	**hCA II**	**hCA IX**	**hCA XII**
**7a**	H	184.6	69.2	8.7	32.5
**7b**	4-Cl	349.0	51.6	35.7	9.1
**7c**	4-OCF_3_	2410	954.2	9.7	158.3
**7d**	2-F	223.7	139.6	14.3	65.4
**7e**	4-CF_3_	853.6	77.5	8.0	9.6
**7f**	4-allyloxy	688.5	320.1	5.6	67.6
**7g**	3,4,5-triOMe	173.0	31.6	4.2	46.0
**7h**	3-EtO-4-OH	22.4	7.3	5.6	63.7
**7i**	4-CN	124.9	56.6	7.3	9.0
**7j**	3-OMe	227.6	46.1	15.8	73.6
**7k**	4-OMe	78.6	12.7	9.3	58.2
**7l**	4-*i*Pr	430.3	68.6	51.5	41.6
**7m**	3-OPh	470.8	55.5	28.2	47.0
**7n**	3,4-diOMe	84.0	15.4	8.1	38.5
**7o**	2,4,5-triOMe	338.9	176.3	34.7	9.5
**7p**	2,4-diOMe	385.6	8.0	17.7	9.8
**7q**	2,4,6-triOMe	298.5	53.1	23.6	26.3
**7r**	3,4-diF	212.7	19.6	13.5	9.6
**7s**	3-NO_2_	77.9	14.6	8.4	9.0
**7t**	4-OH-3,5-diOMe	77.5	9.6	7.2	30.2
**7u**	4-NO_2_	70.6	8.9	7.7	9.5
**9a**	H	41.6	4.3	4.7	56.2
**9b**	5-Me	92.2	5.5	8.9	40.6
**9c**	5-CF_3_	319.2	67.8	13.3	37.8
**9d**	5-Cl	35.8	5.2	6.0	26.3
**AAZ**		250.0	12.1	25.8	5.7

* The mean from three different assays using a stopped flow technique. Errors were in the range of ± 5–10% of the reported values.

## Data Availability

Data are available within the manuscript.

## Supplementary Materials

The following supporting information can be downloaded at: https://www.mdpi.com/article/10.3390/molecules27228028/s1, Figures S1–S54: ^1^H and ^13^C NMR spectra of intermediates and final compounds.

## Sample Availability

Samples of the compounds are available from the authors upon request.

## References

[1] Krátký M., Štěpánková Š., Vorčáková K., Vinšová J. (2016). Synthesis and in vitro evaluation of novel rhodanine derivatives as potential cholinesterase inhibitors. Bioorg Chem..

[2] Patel A.B., Kumari P., Ravi V. (2016). Scope of Selective Heterocycles from Organic and Pharmaceutical Perspective: Recent Advances in the Biological Importance of Rhodanine Derivatives.

[3] Mermer A. (2021). The Importance of Rhodanine Scaffold in Medicinal Chemistry: A Comprehensive Overview. Mini Rev. Med. Chem..

[4] Mousavi S.M., Zarei M., Hashemi S.A., Babapoor A., Amani A.M. (2019). A conceptual review of rhodanine: Current applications of antiviral drugs, anticancer and antimicrobial activities. Artif. Cells Nanomed. Biotechnol..

[5] Szczepański J., Tuszewska H., Trotsko N. (2022). Anticancer Profile of Rhodanines: Structure-Activity Relationship (SAR) and Molecular Targets-A Review. Molecules.

[6] Maddila S., Gorle S., Jonnalagadda S.B. (2020). Drug screening of rhodanine derivatives for antibacterial activity. Expert Opin. Drug Discov..

[7] Bayindir S., Caglayan C., Karaman M., Gülcin İ. (2019). The green synthesis and molecular docking of novel N-substituted rhodanines as effective inhibitors for carbonic anhydrase and acetylcholinesterase enzymes. Bioorg. Chem..

[8] Kaminskyy D., Kryshchyshyn A., Lesyk R. (2017). Recent developments with rhodanine as a scaffold for drug discovery. Expert Opin. Drug Discov..

[9] Mermer A., Demirbas N., Colak A., Demir E.A., Kulabas N., Demirbas A. (2018). One-pot, Four-Component Green Synthesis, Carbonic Anhydrase II Inhibition and Docking Studies of 5-Arylidenerhodanines. Chem. Select..

[10] Yin L.J., Bin Ahmad Kamar A.K.D., Fung G.T., Liang C.T., Avupati V.R. (2022). Review of anticancer potentials and structure-activity relationships (SAR) of rhodanine derivatives. Biomed. Pharmacother..

[11] Swain B., Sahoo S.K., Singh P., Angeli A., Yaddanapudi V.M., Supuran C.T., Arifuddin M. (2022). Exploration of 2-phenylquinoline-4-carboxamide linked benzene sulfonamide derivatives as isoform selective inhibitors of transmembrane human carbonic anhydrases. Eur. J. Med. Chem..

[12] Capasso C., Supuran C.T. (2013). Anti-infective carbonic anhydrase inhibitors: A patent and literature review. Expert Opin. Ther. Pat..

[13] Pan P., Vermelho A.B., Scozzafava A., Parkkila S., Capasso C., Supuran C.T. (2013). Anion inhibition studies of the α-carbonic anhydrase from the protozoan pathogen Trypanosoma cruzi, the causative agent of Chagas disease. Bioorganic Med. Chem..

[14] Del Prete S., Vullo D., Fisher G.M., Andrews K.T., Poulsen S.A., Capasso C., Supuran C.T. (2014). Discovery of a new family of carbonic anhydrases in the malaria pathogen Plasmodium falciparum--the η-carbonic anhydrases. Bioorganic Med. Chem. Lett..

[15] Supuran C.T., Capasso C. (2015). The η-class carbonic anhydrases as drug targets for antimalarial agents. Expert Opin. Ther. Targets.

[16] Güzel-Akdemir Ö., Akdemir A., Pan P., Vermelho A.B., Parkkila S., Scozzafava A., Capasso C., Supuran C.T. (2013). A class of sulfonamides with strong inhibitory action against the α-carbonic anhydrase from Trypanosoma cruzi. J. Med. Chem..

[17] Arechederra R.L., Waheed A., Sly W.S., Supuran C.T., Minteer S.D. (2013). Effect of sulfonamides as carbonic anhydrase VA and VB inhibitors on mitochondrial metabolic energy conversion. Bioorganic Med. Chem..

[18] McDonald P.C., Chafe S.C., Supuran C.T., Dedhar S. (2022). Cancer Therapeutic Targeting of Hypoxia Induced Carbonic Anhydrase IX: From Bench to Bedside. Cancers.

[19] Deniz S., Uysal T.K., Capasso C., Supuran C.T., Ozensoy Guler O. (2021). Is carbonic anhydrase inhibition useful as a complementary therapy of Covid-19 infection?. J. Enzym. Inhib. Med. Chem..

[20] Aggarwal M., McKenna R. (2012). Update on carbonic anhydrase inhibitors: A patent review (2008–2011). Expert Opin. Ther. Pat..

[21] Touisni N., Maresca A., McDonald P.C., Lou Y., Scozzafava A., Dedhar S., Winum J.Y., Supuran C.T. (2011). Glycosyl coumarin carbonic anhydrase IX and XII inhibitors strongly attenuate the growth of primary breast tumors. J. Med. Chem..

[22] Scozzafava A., Supuran C.T., Carta F. (2013). Antiobesity carbonic anhydrase inhibitors: A literature and patent review. Expert Opin. Ther. Pat..

[23] Fabrizi F., Mincione F., Somma T., Scozzafava G., Galassi F., Masini E., Impagnatiello F., Supuran C.T. (2012). A new approach to antiglaucoma drugs: Carbonic anhydrase inhibitors with or without NO donating moieties. Mechanism of action and preliminary pharmacology. J. Enzym. Inhib. Med. Chem..

[24] Appetecchia F., Consalvi S., Berrino E., Gallorini M., Granese A., Campestre C., Carradori S., Biava M., Poce G. (2021). A Novel Class of Dual-Acting DCH-CORMs Counteracts Oxidative Stress-Induced Inflammation in Human Primary Tenocytes. Antioxidants.

[25] Singh P., Purnachander Yadav P., Swain B., Thacker P.S., Angeli A., Supuran C.T., Arifuddin M. (2021). Discovery of a novel series of indolylchalcone-benzenesulfonamide hybrids acting as selective carbonic anhydrase II inhibitors. Bioorganic Chem..

[26] Singh P., Sridhar Goud N., Swain B., Kumar Sahoo S., Choli A., Angeli A., Singh Kushwah B., Madhavi Yaddanapudi V., Supuran C.T., Arifuddin M. (2022). Synthesis of a new series of quinoline/pyridine indole-3-sulfonamide hybrids as selective carbonic anhydrase IX inhibitors. Bioorganic Med. Chem. Lett..

[27] Elkamhawy A., Woo J., Nada H., Angeli A., Bedair T.M., Supuran C.T., Lee K. (2022). Identification of Novel and Potent Indole-Based Benzenesulfonamides as Selective Human Carbonic Anhydrase II Inhibitors: Design, Synthesis, In Vitro, and In Silico Studies. Int. J. Mol. Sci..

[28] Singh P., Choli A., Swain B., Angeli A., Sahoo S.K., Yaddanapudi V.M., Supuran C.T., Arifuddin M. (2022). Design and development of novel series of indole-3-sulfonamide ureido derivatives as selective carbonic anhydrase II inhibitors. Arch. Pharm..

[29] Demir-Yazıcı K., Bua S., Akgüneş N.M., Akdemir A., Supuran C.T., Güzel-Akdemir Ö. (2019). Indole-Based Hydrazones Containing A Sulfonamide Moiety as Selective Inhibitors of Tumor-Associated Human Carbonic Anhydrase Isoforms IX and XII. Int. J. Mol. Sci..

[30] Żeslawska E., Nitek W., Tejchman W. (2015). The Synthesis and Crystal Structures of the Homologues of Epalrestat. J. Chem. Crystallogr..

[31] Negah S., Mehdi K., Mohsen A., Amirhossein S., Hamid N., Alireza M., Saeed E., Ebrahim S.M., Alireza F., Abbas S. (2015). Synthesis and biological evaluation of 5-benzylidenerhodanine-3-acetic acid derivatives as AChE and 15-LOX inhibitors. J. Enzym. Inhib. Med. Chem..

[32] Bourahla K., Guihéneuf S., Limanton E., Paquin L., Le Guével R., Charlier T., Rahmouni M., Durieu E., Lozach O., Carreaux F. (2021). Design and Microwave Synthesis of New (5Z) 5-Arylidene-2-thioxo-1,3-thiazolinidin-4-one and (5Z) 2-Amino-5-arylidene-1,3-thiazol-4(5H)-one as New Inhibitors of Protein Kinase DYRK1A. Pharmaceuticals.

[33] Khalifah R.G. (1971). The carbon dioxide hydration activity of carbonic anhydrase. I. Stop-flow kinetic studies on the native human isoenzymes B and C. J. Biol. Chem..

[34] Liguori F., Carradori S., Ronca R., Rezzola S., Filiberti S., Carta F., Turati M., Supuran C.T. (2022). Benzenesulfonamides with different rigidity-conferring linkers as carbonic anhydrase inhibitors: An insight into the antiproliferative effect on glioblastoma, pancreatic, and breast cancer cells. J. Enzym. Inhib. Med. Chem..

[35] Güzel-Akdemir Ö., Demir-Yazıcı K., Vullo D., Supuran C.T., Akdemir A. (2022). New Pyridinium Salt Derivatives of 2-(Hydrazinocarbonyl)-3-phenyl-1H-indole-5-sulfonamide as Selective Inhibitors of Tumour-Related Human Carbonic Anhydrase Isoforms IX and XII. Anti-Cancer Agents Med. Chem..

[36] Supuran C.T., Clare B.W. (1999). Carbonic anhydrase inhibitors—Part 57: Quantum chemical QSAR of a group of 1,3,4-thiadiazole- and 1,3,4-thiadiazoline disulfonamides with carbonic anhydrase inhibitory properties. Eur. J. Med. Chem..

[37] Carta F., Supuran C.T., Scozzafava A. (2014). Sulfonamides and their isosters as carbonic anhydrase inhibitors. Future Med. Chem..

