# Supplementary material for: Design, Synthesis and Biological Assessment of Rhodanine-Linked Benzenesulfonamide Derivatives as Selective and Potent Human Carbonic Anhydrase Inhibitors
Source: Molecules. 2022 Nov 18;27(22):8028. doi: 10.3390/molecules27228028 (PMC9697818; doi:10.3390/molecules27228028)
Supplement: Supplementary file 1 [file molecules-27-08028-s001.zip › molecules-2029220-supplementary.pdf]

**Supporting information**

# Design, synthesis and biological assessment of rhodanine-linked benzenesulfonamide derivatives as selective and potent human carbonic anhydrase inhibitors

Baijayantimala Swain[1], Abrar Khan[1], Priti Singh[1], Vaibhav S. Marde[1], Andrea Angeli[2], Krishna Kartheek Chinchilli[1], Venkata Madhavi Yaddanapudi[1], Simone Carradori[3,*], Claudiu T. Supuran[2] and Mohammed Arifuddin[1,4]

[1]Department of Chemical Sciences National Institute of Pharmaceutical Education and Research (NIPER), Balanagar, Hyderabad 500037, India

[2]Neurofarba Department, University of Florence, Via Ugo Schiff 6, 50019 Sesto Fiorentino, Florence, Italy

[3]Department of Pharmacy, "G. d'Annunzio" University of Chieti-Pescara, via dei Vestini 31, Chieti 66100, Italy

[4]Department of Chemistry, Directorate of Distance Education, Maulana Azad National Urdu University, Gachibowli, Hyderabad 500032, Telangana, India

***Correspondence:** Prof. Simone Carradori, simone.carradori@unich.it

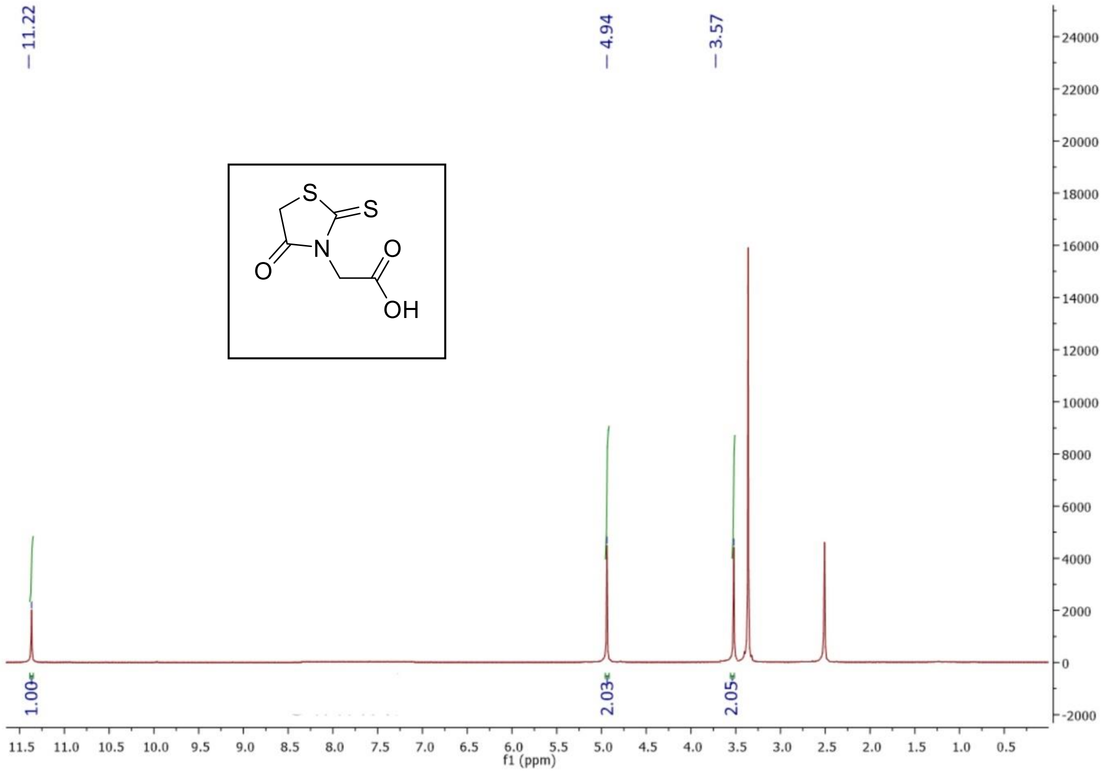


Figure S1. $^{1}H$ NMR of **3** in DMSO-$d_6$

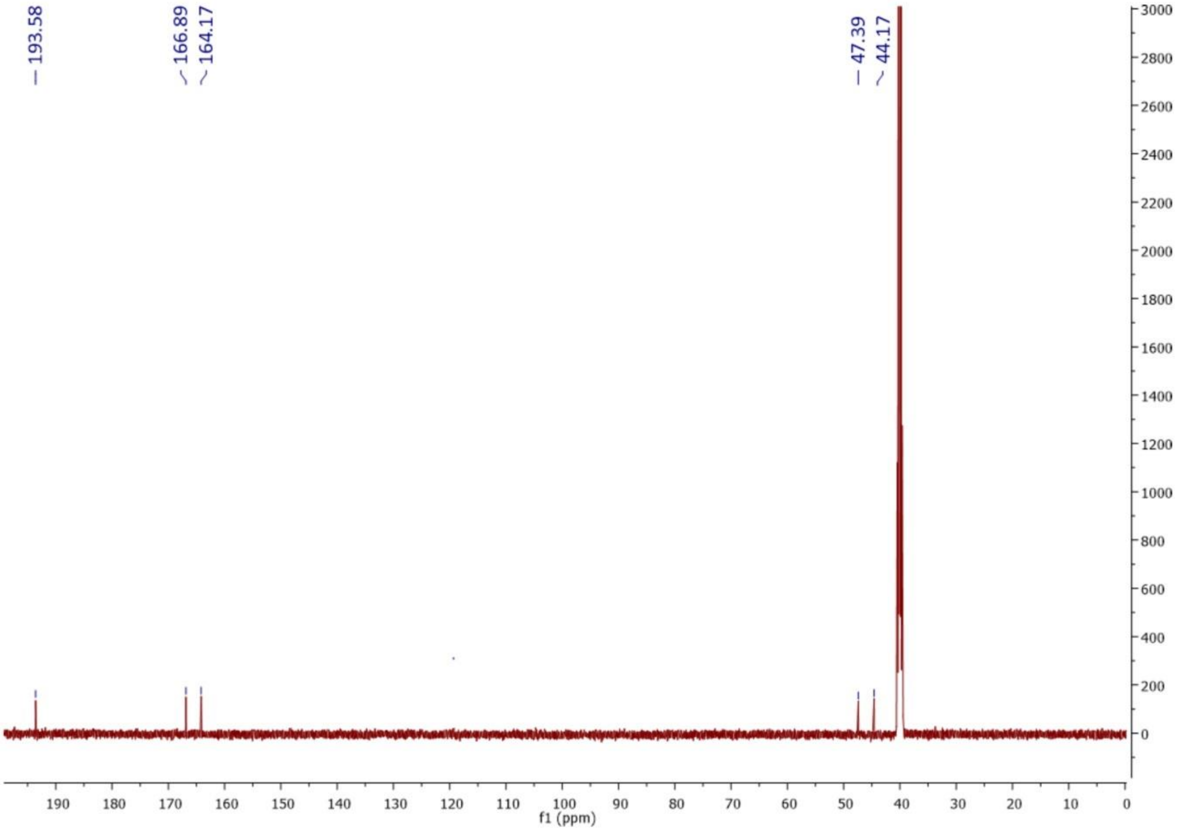


Figure S2. $^{13}C$ NMR of **3** in DMSO-$d_6$

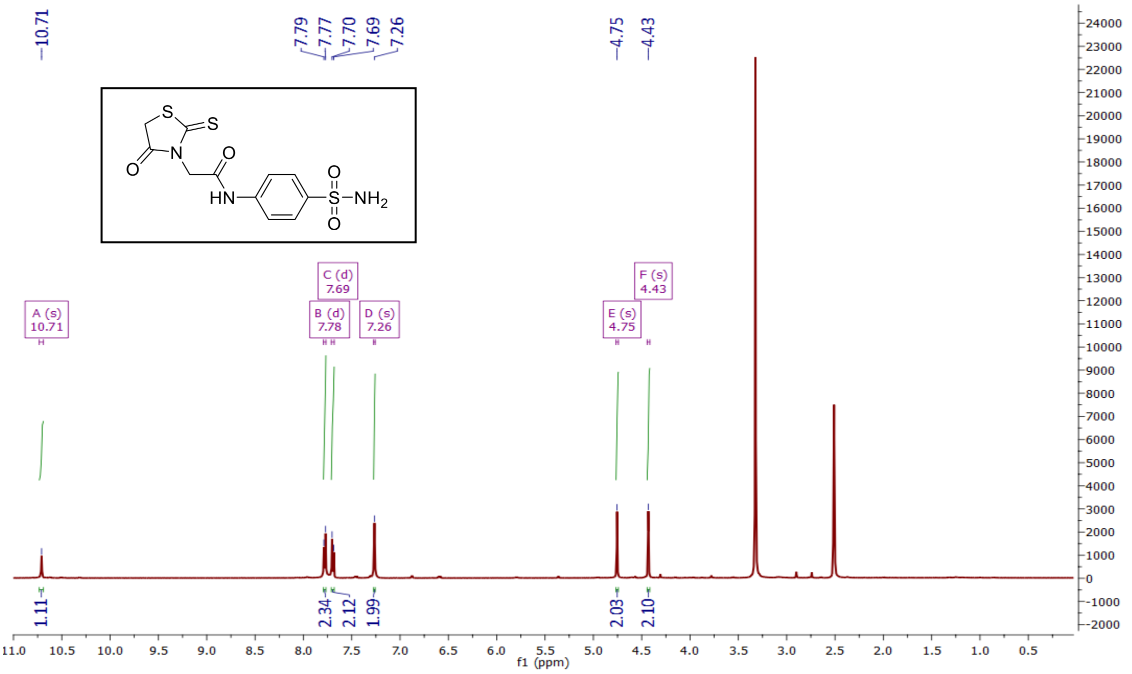


Figure S3. $^{1}$H NMR of **5** in DMSO-$d_6$

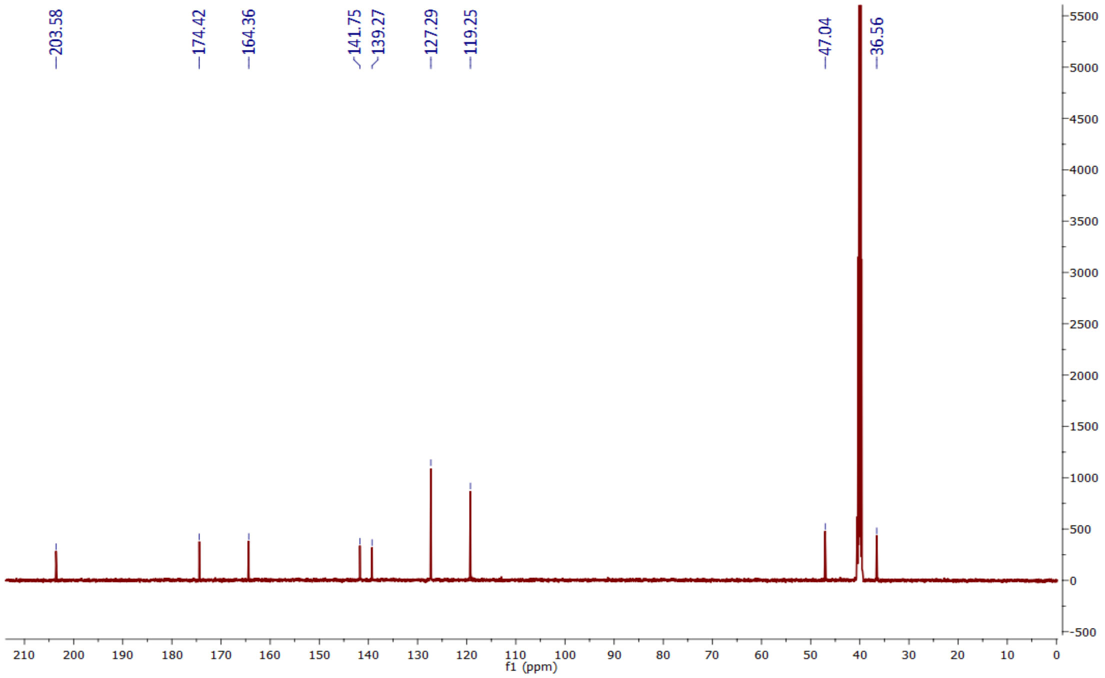


Figure S4. $^{13}$C NMR of **5** in DMSO-$d_6$

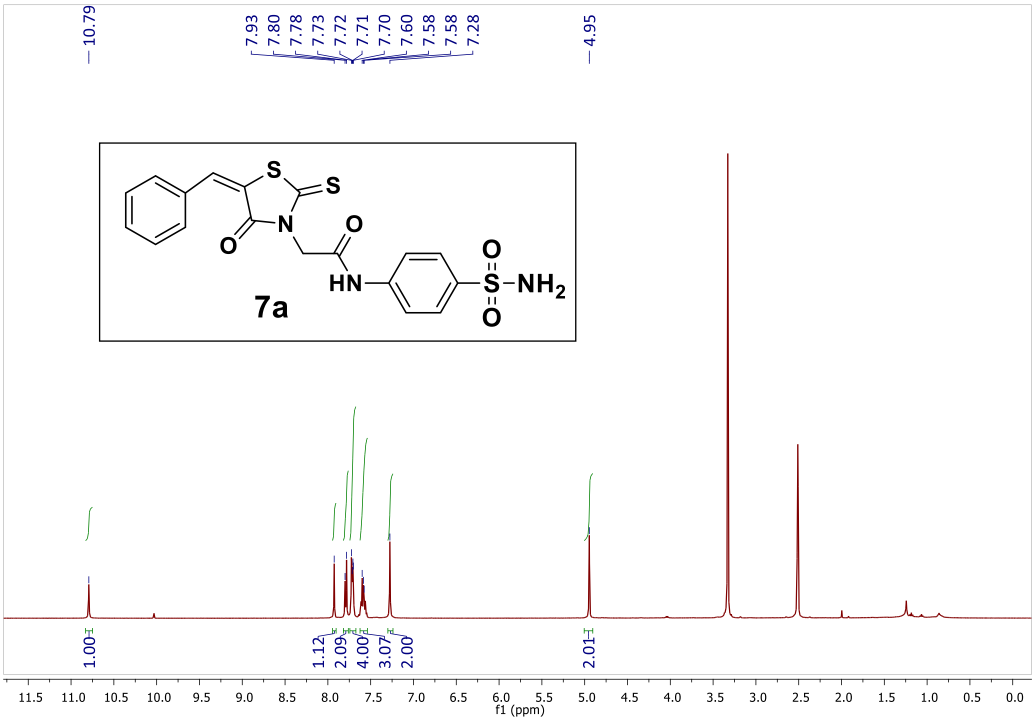


Figure S5. $^{1}$H NMR of **7a** in DMSO-$d_6$

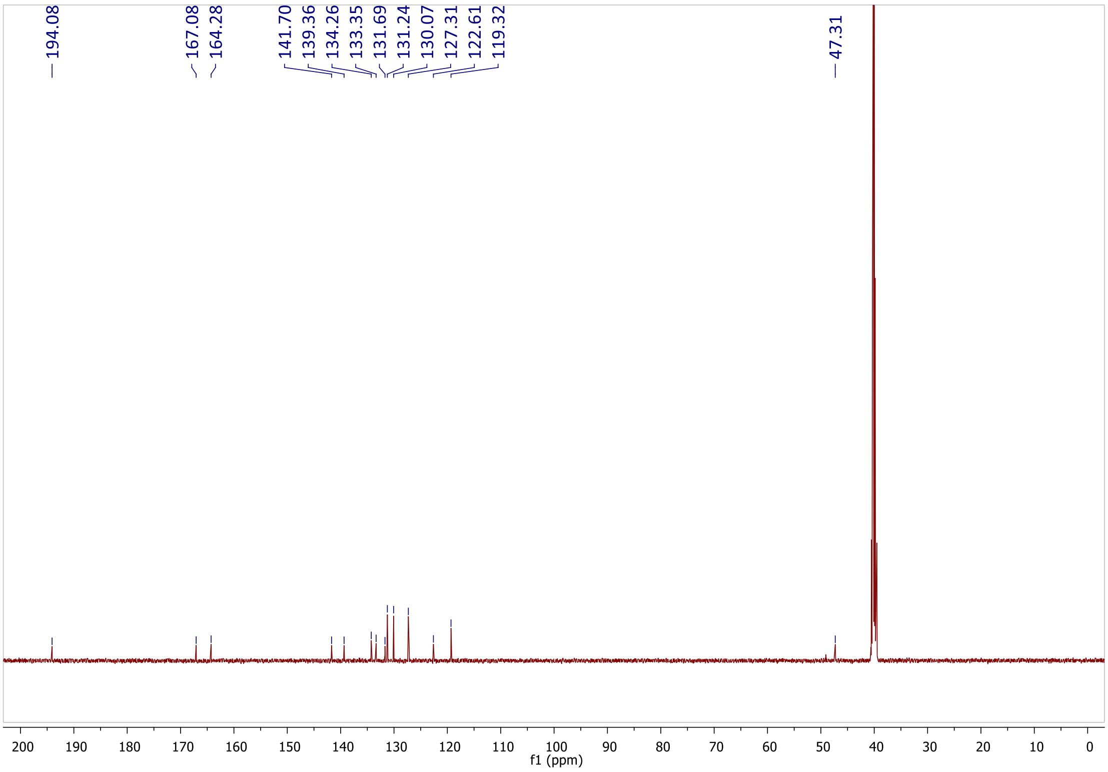


Figure S6. $^{13}$C NMR of **7a** in DMSO-$d_6$

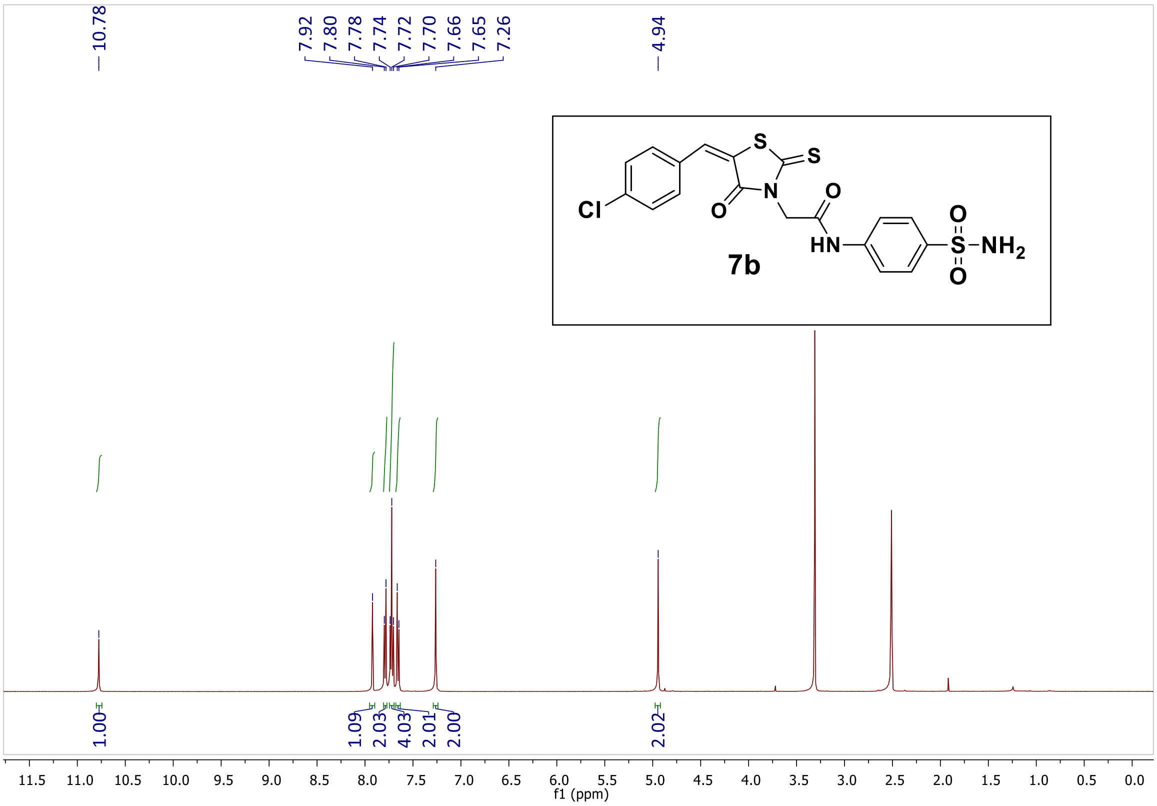


Figure S7. $^{1}$H NMR of **7b** in DMSO-*d*$_6$

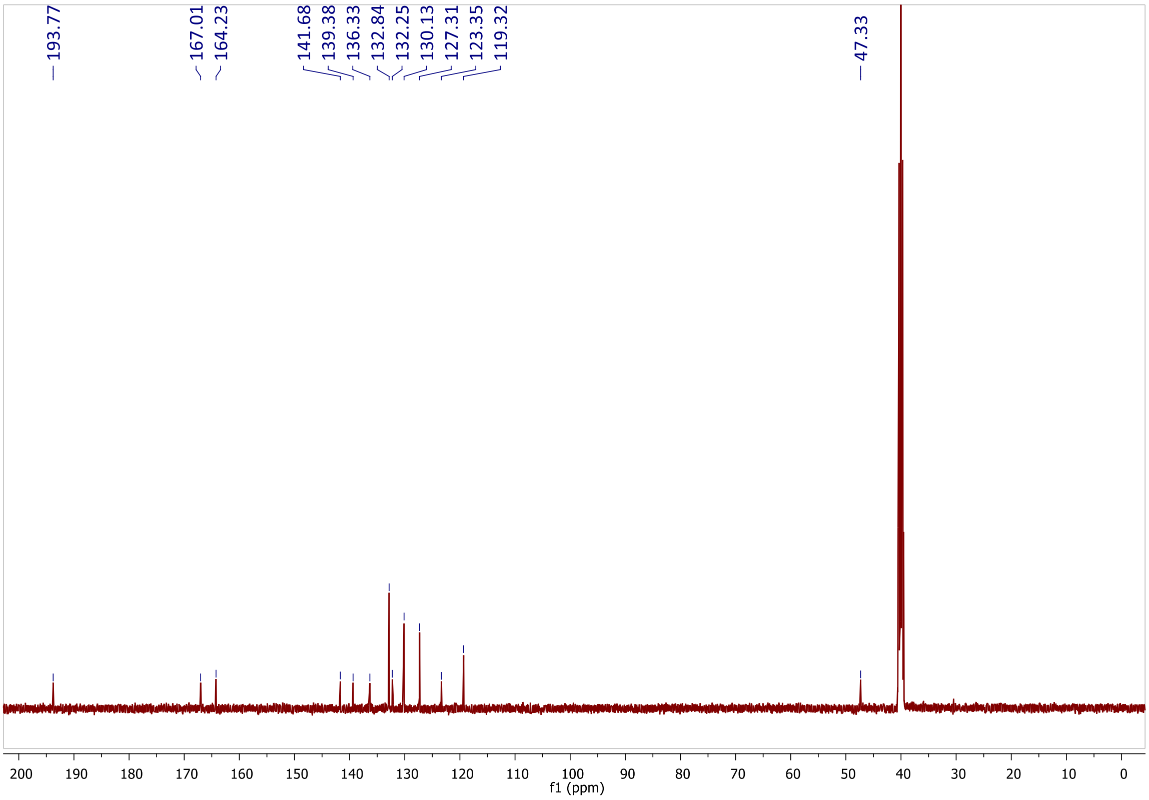


Figure S8. $^{13}$C NMR of **7b** in DMSO-*d*$_6$

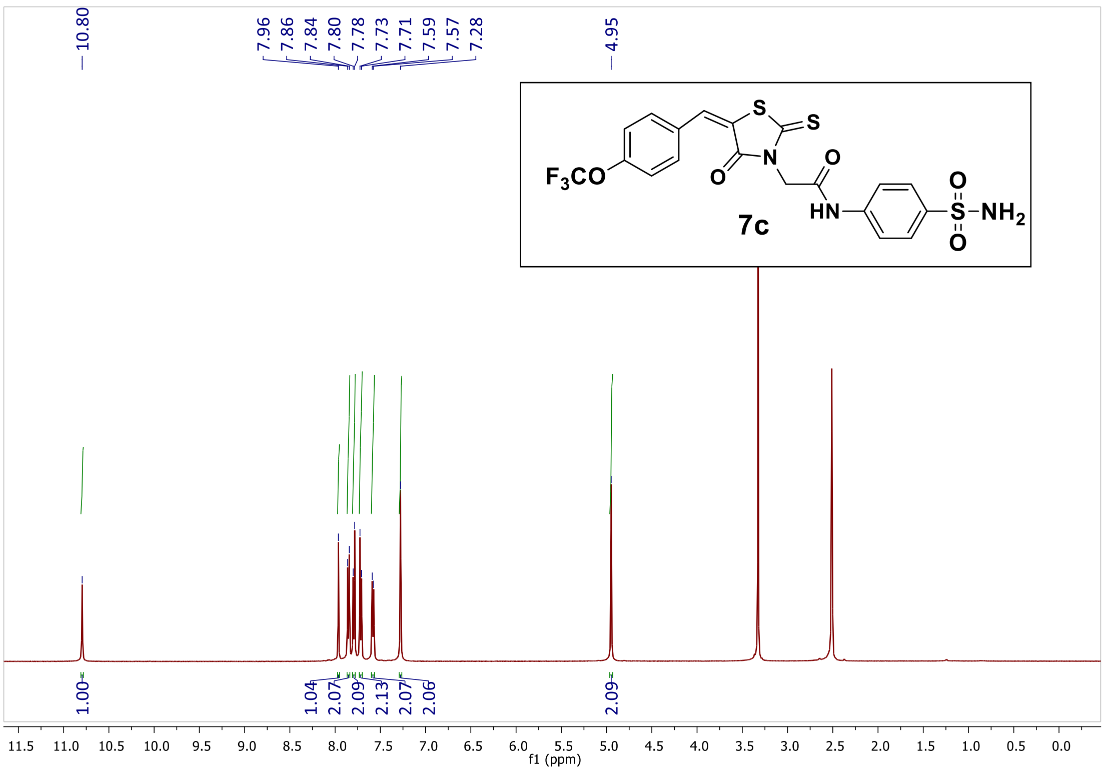


Figure S9. $^{1}$H NMR of **7c** in DMSO-*d*6

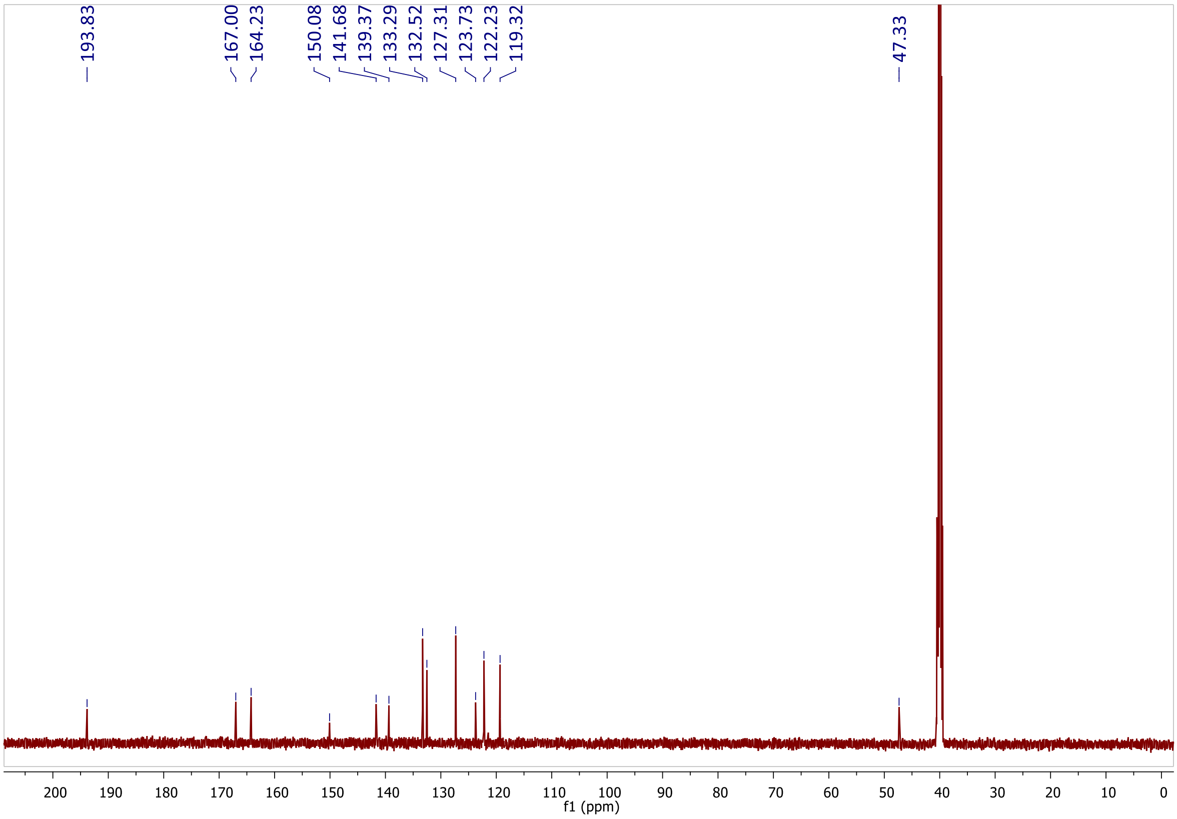


Figure S10. $^{13}$C NMR of **7c** in DMSO-*d*6

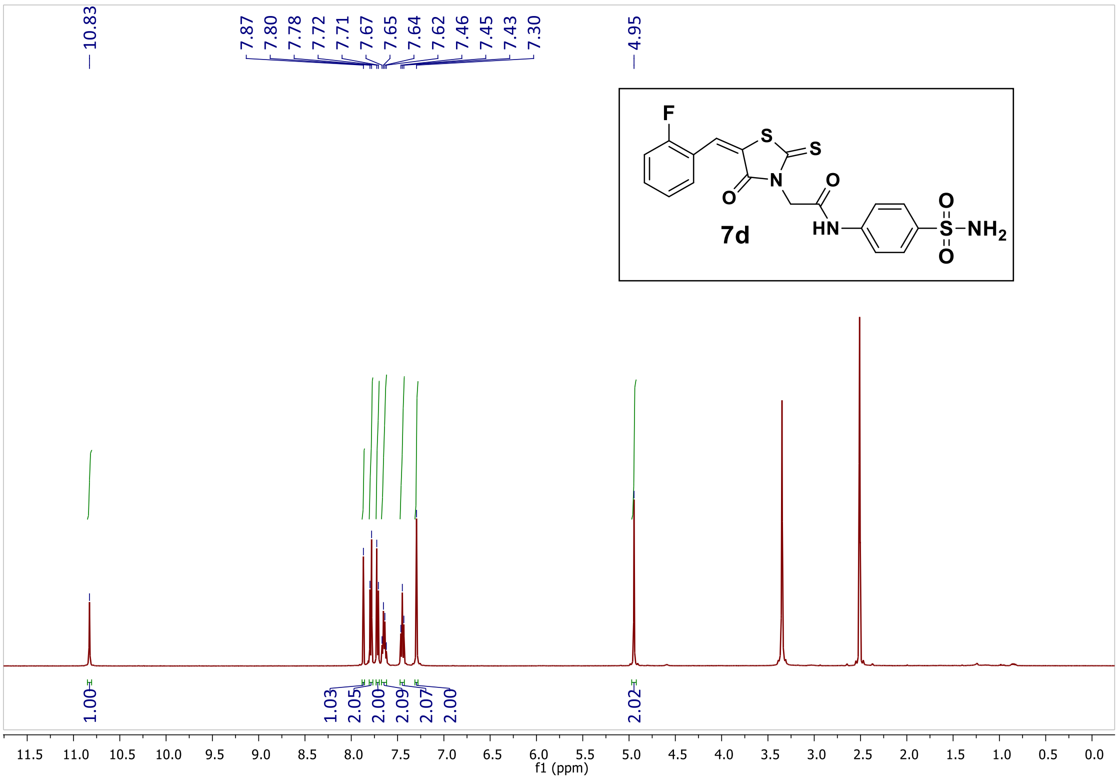


Figure S11. $^{1}$H NMR of **7d** in DMSO-$d_6$

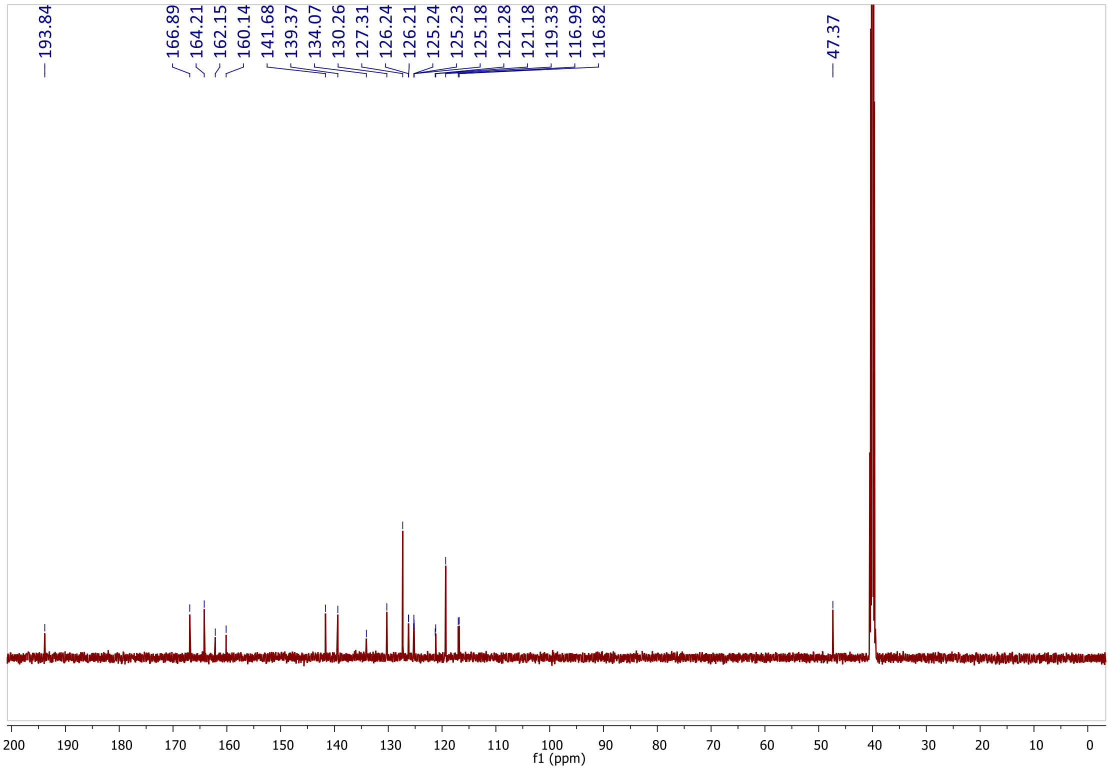


Figure S12. $^{13}$C NMR of **7d** in DMSO-$d_6$

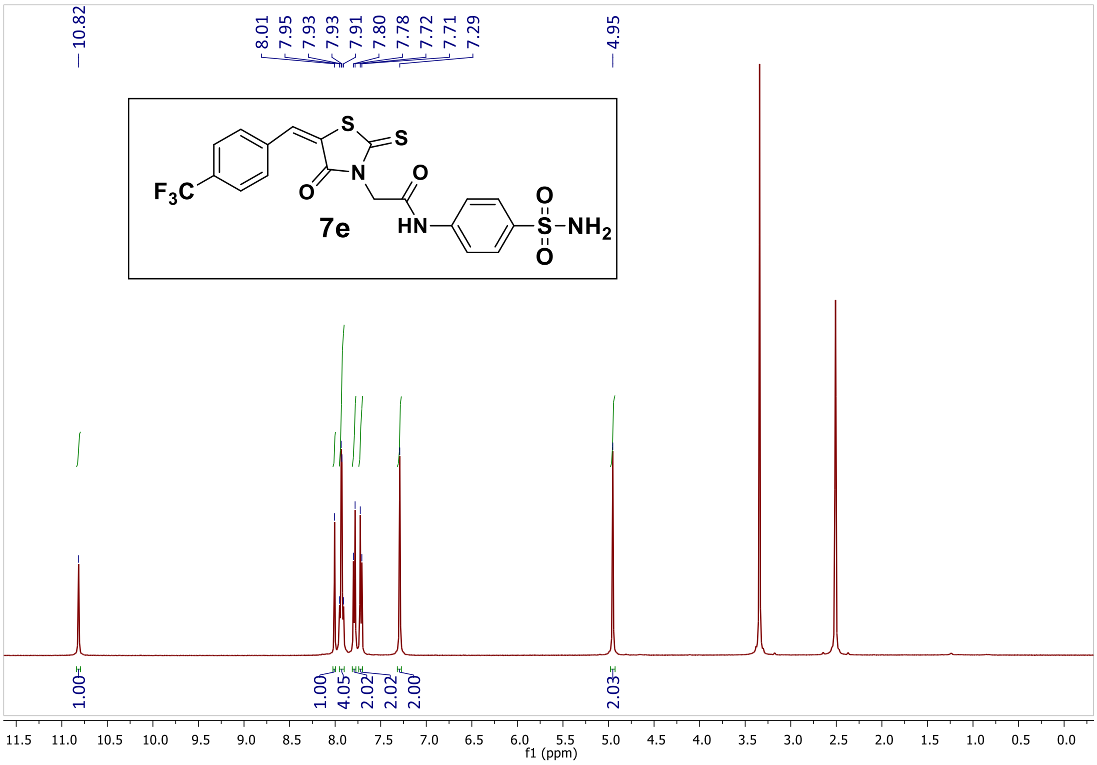


Figure S13. $^{1}$H NMR of **7e** in DMSO-*d*6

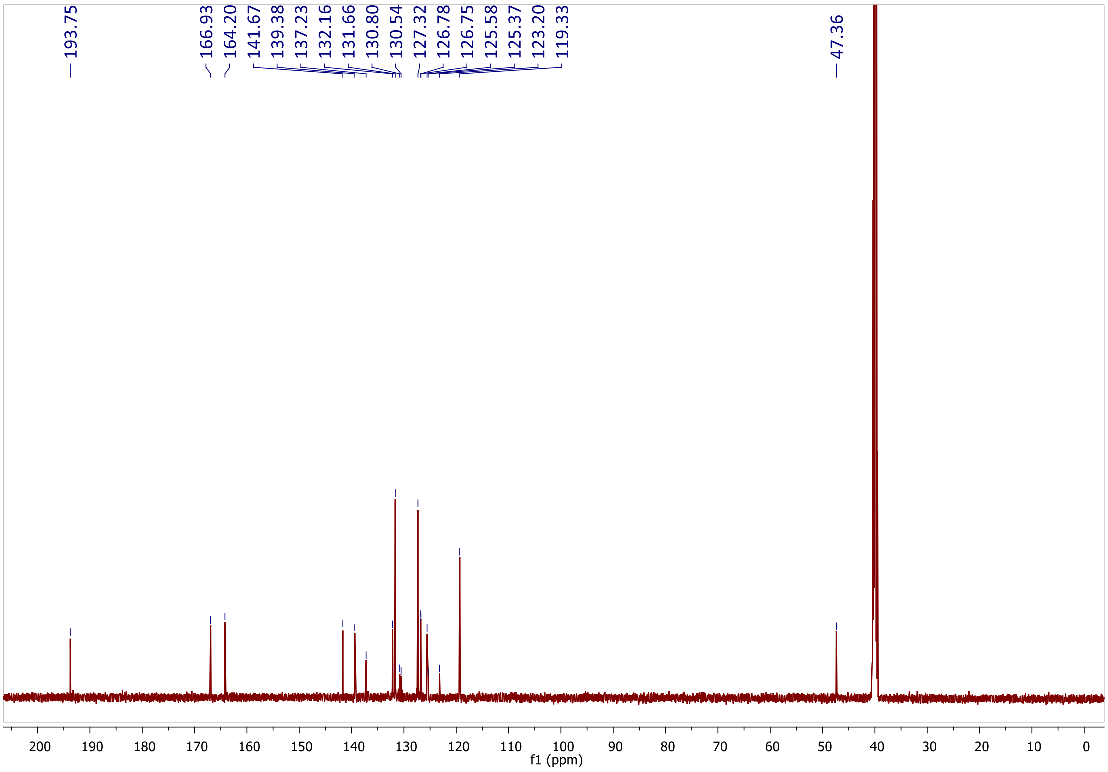


Figure S14. $^{13}$C NMR of **7e** in DMSO-*d*6

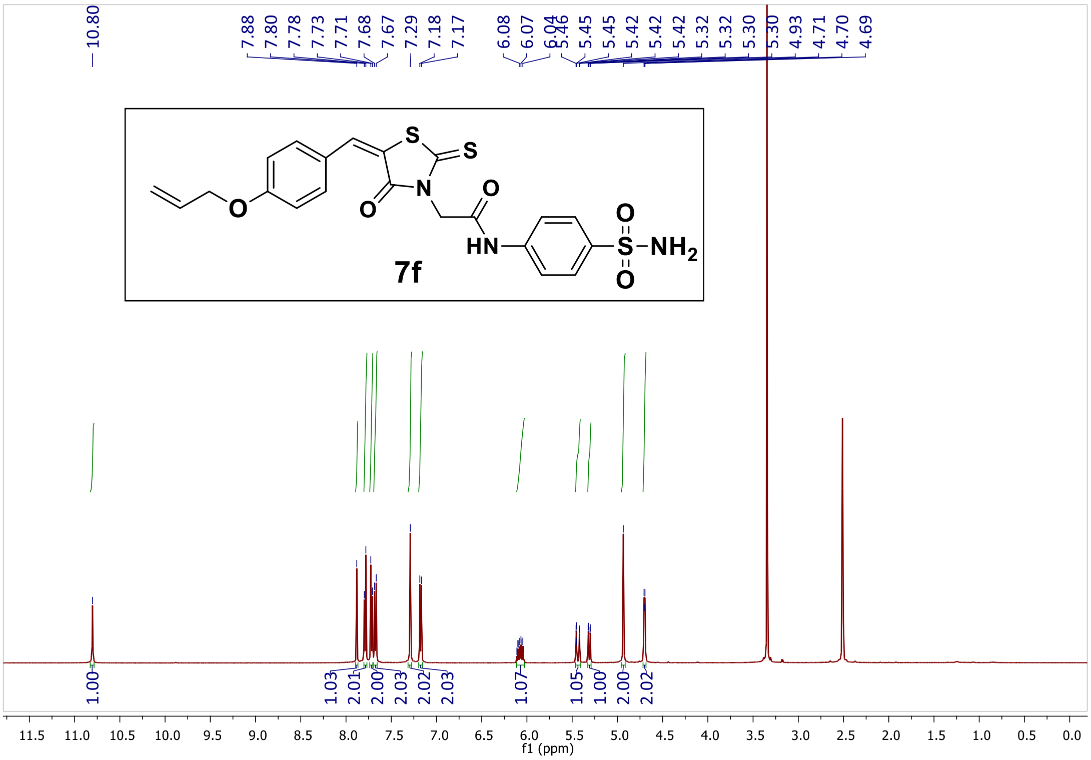


Figure S15. $^{1}H$ NMR of **7f** in DMSO-*d*6

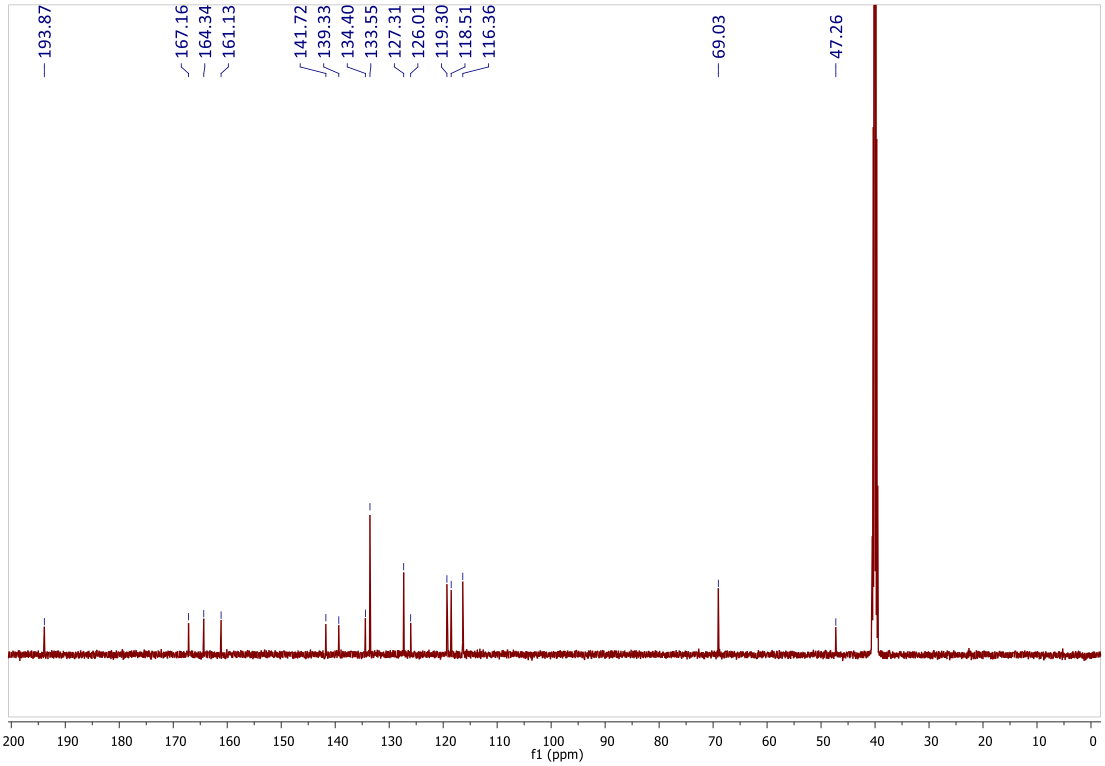


Figure S16. $^{13}C$ NMR of **7f** in DMSO-*d*6

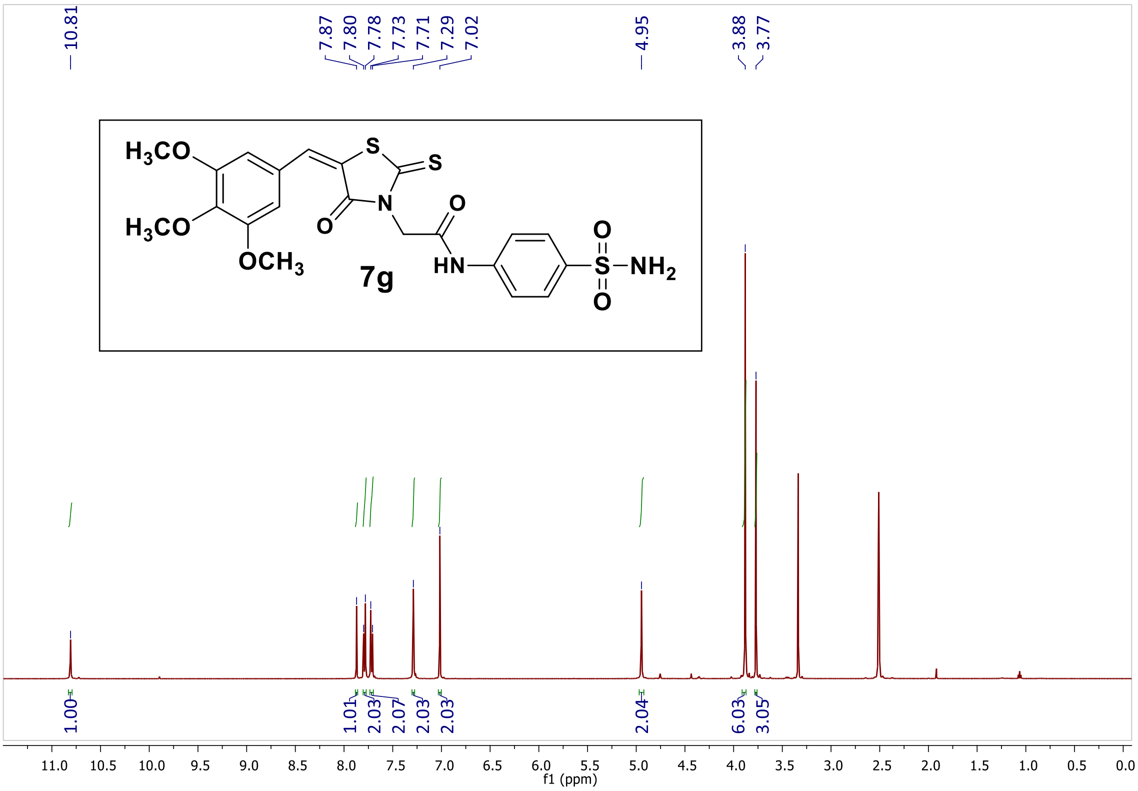


Figure S17. $^{1}$H NMR of **7g** in DMSO-$d_6$

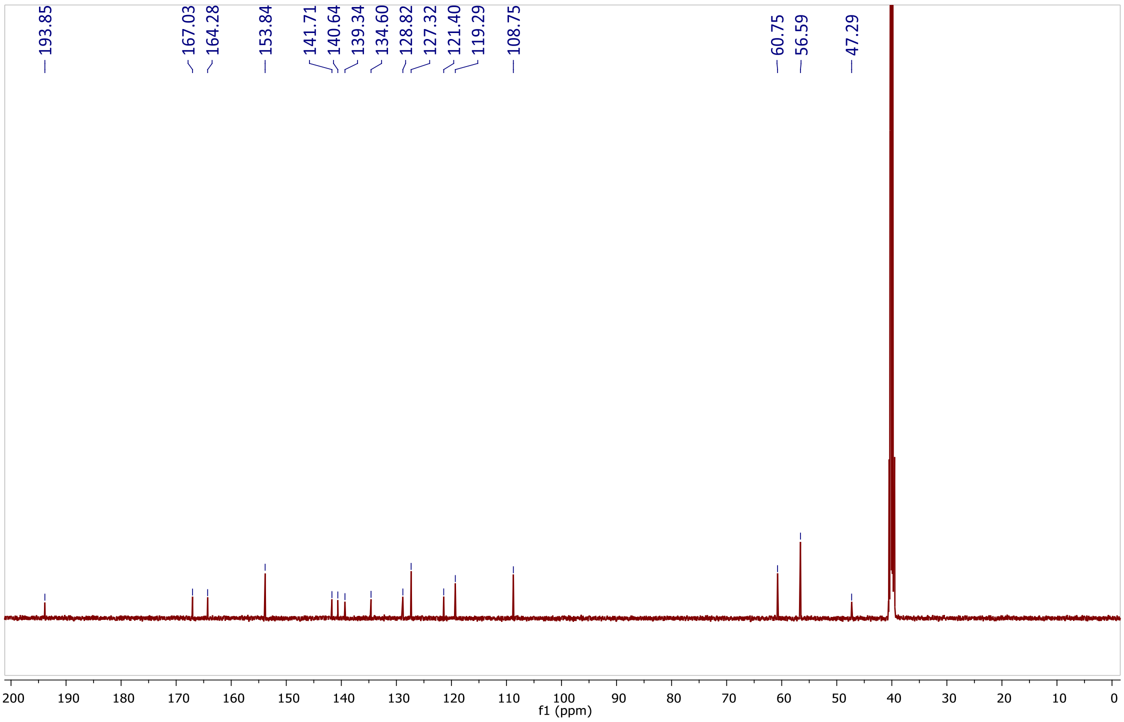


Figure S18. $^{13}$C NMR of **7g** in DMSO-$d_6$

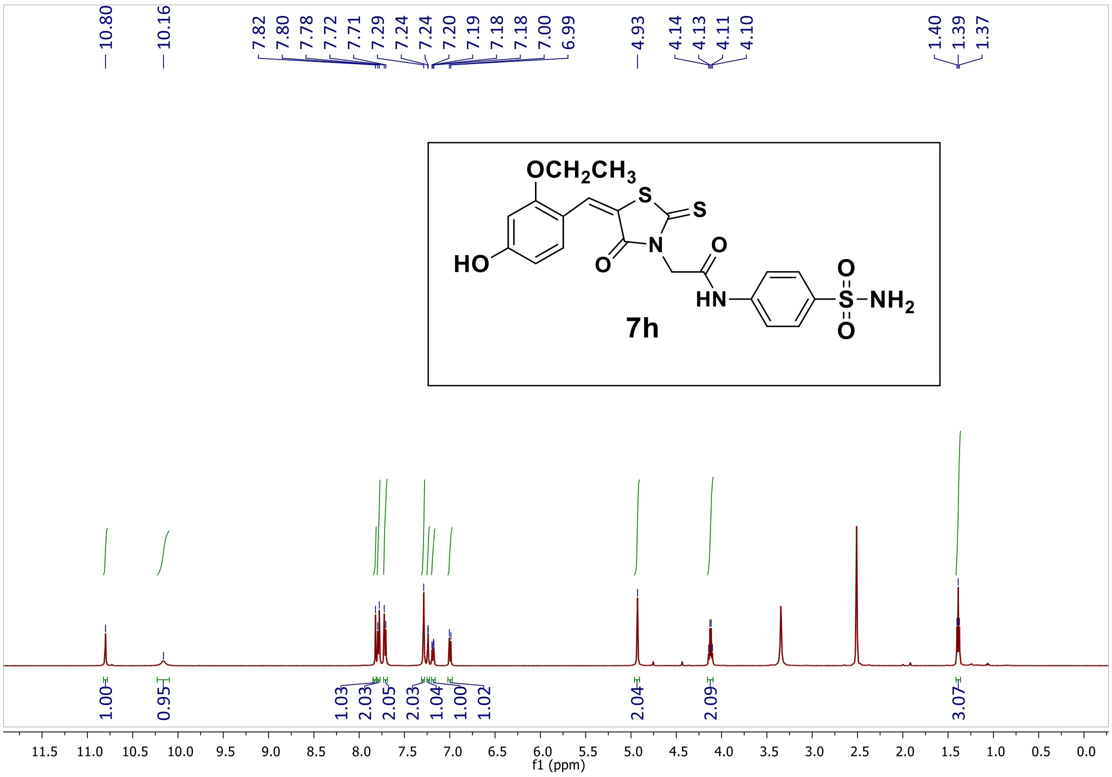


Figure S19. $^{1}$H NMR of **7h** in DMSO-$d_6$

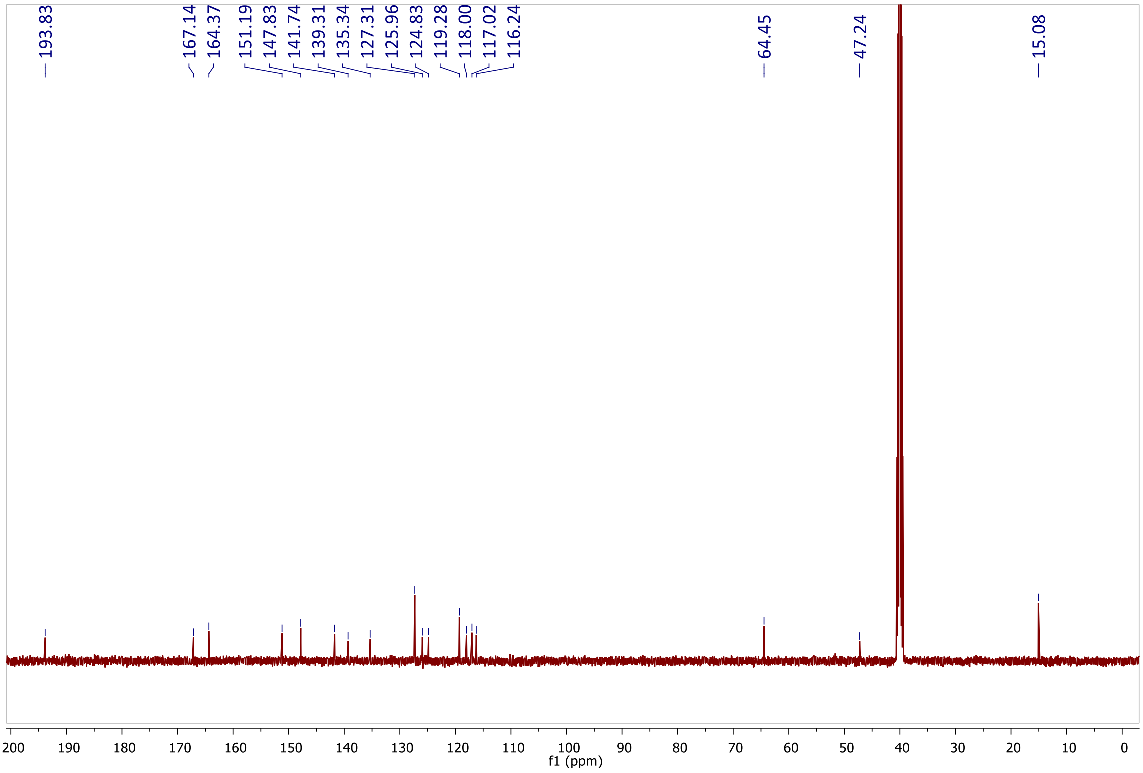


Figure S20. $^{13}$C NMR of **7h** in DMSO-$d_6$

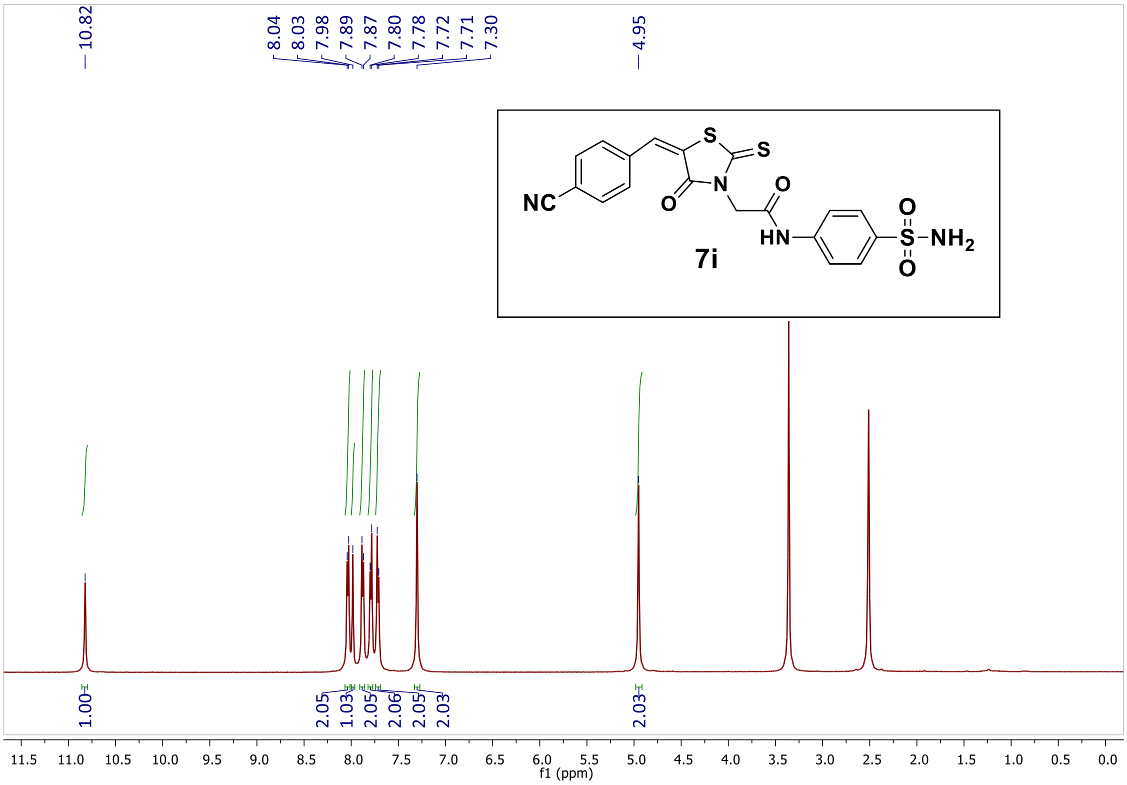


Figure S21. ${}^{1}$H NMR of **7i** in DMSO-$d_6$

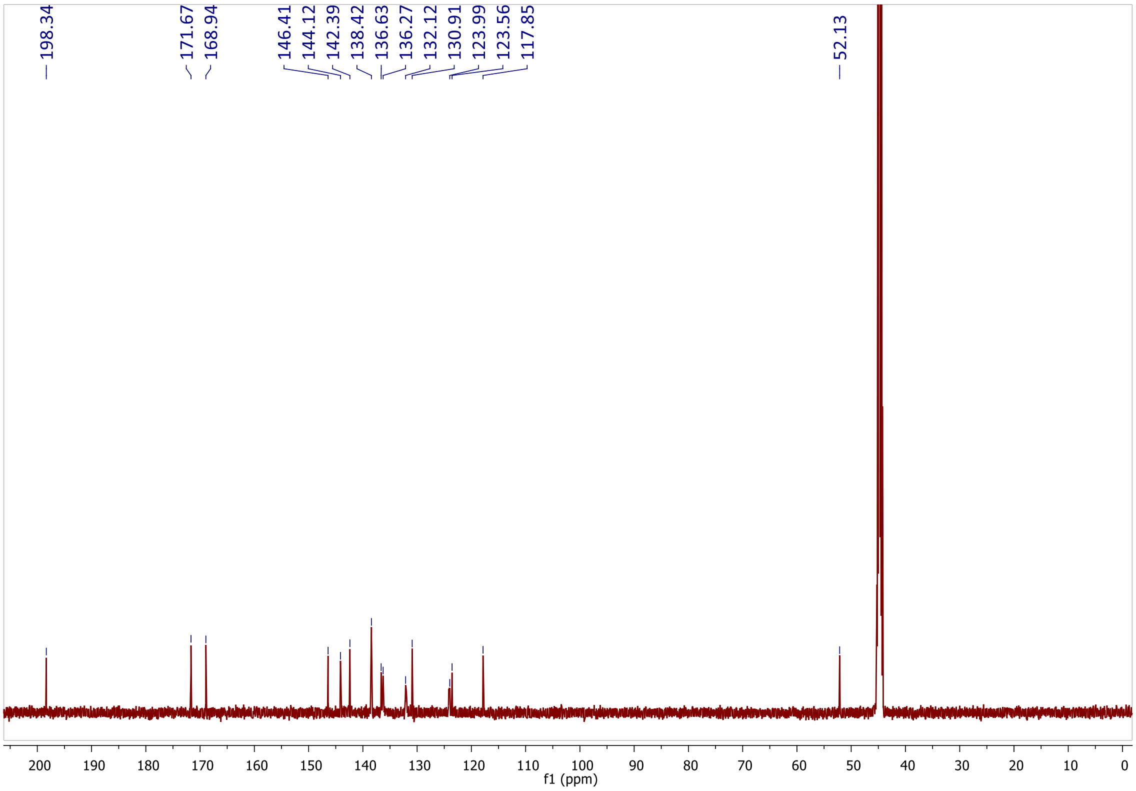


Figure S22. ${}^{13}$C NMR of **7i** in DMSO-$d_6$

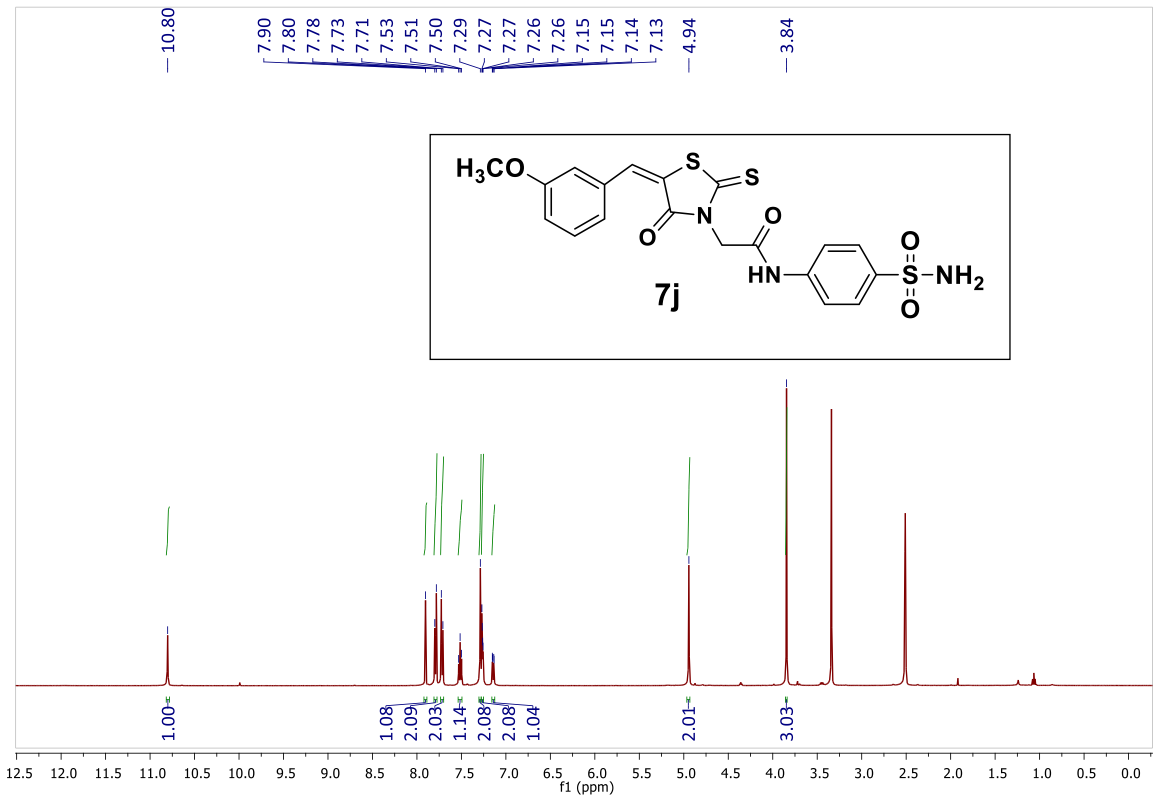


Figure S23. $^{1}$H NMR of **7j** in DMSO-*d*6

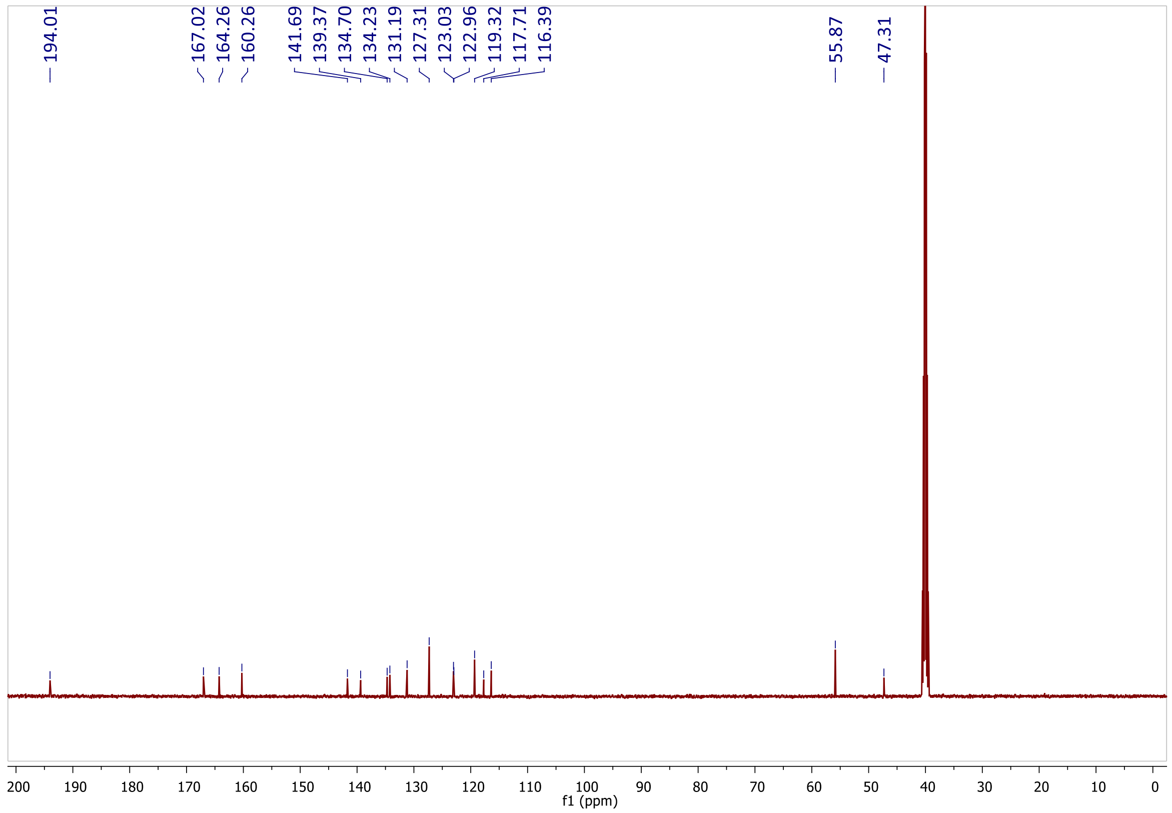


Figure S24. $^{13}$C NMR of **7j** in DMSO-*d*6

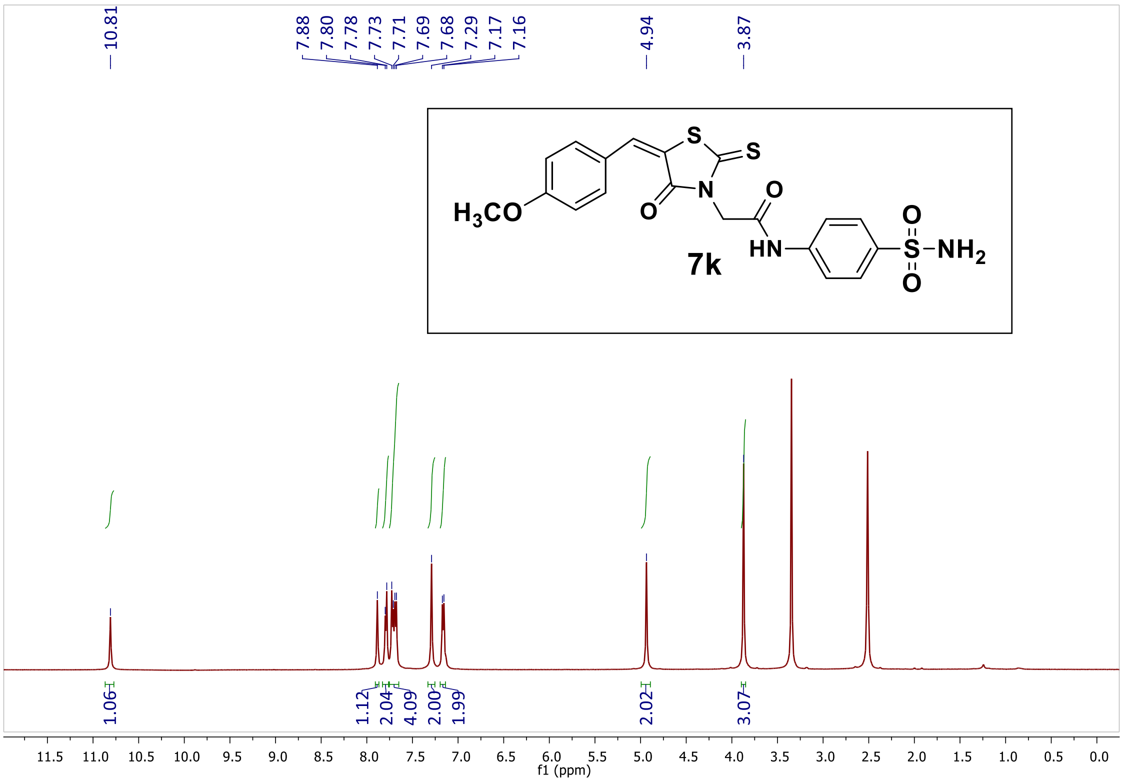


Figure S25. $^{1}$H NMR of **7k** in DMSO-$d_6$

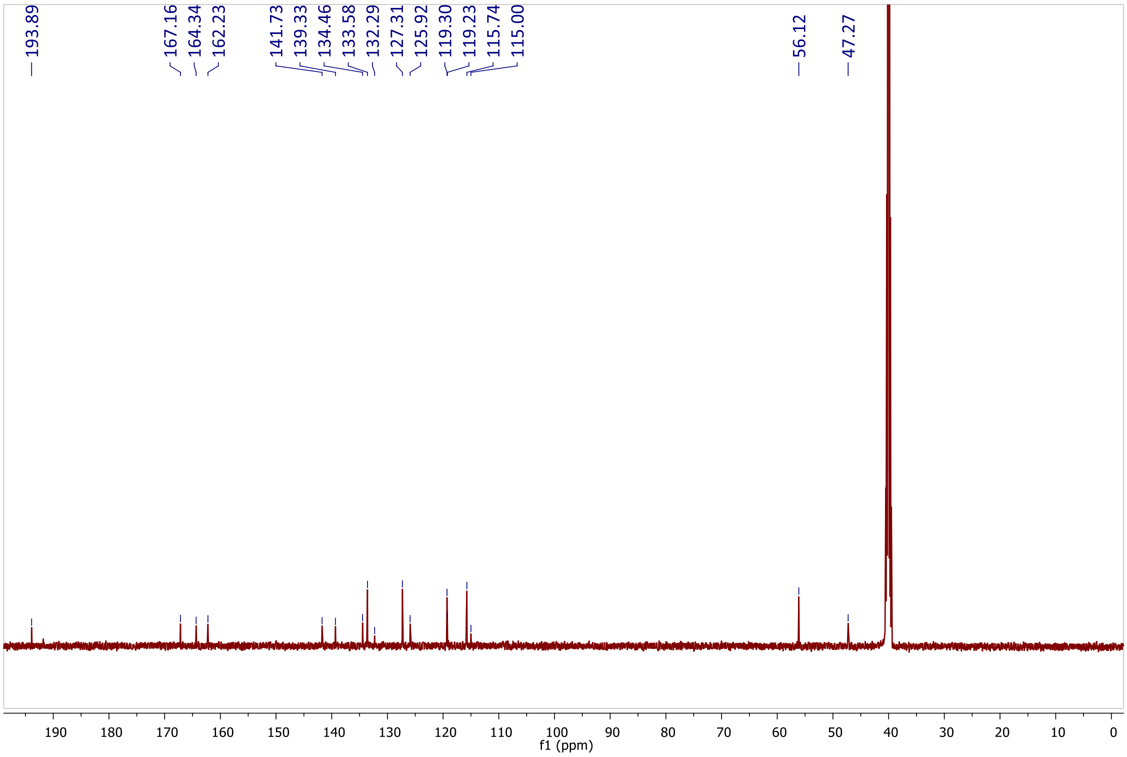


Figure S26. $^{13}$C NMR of **7k** in DMSO-$d_6$

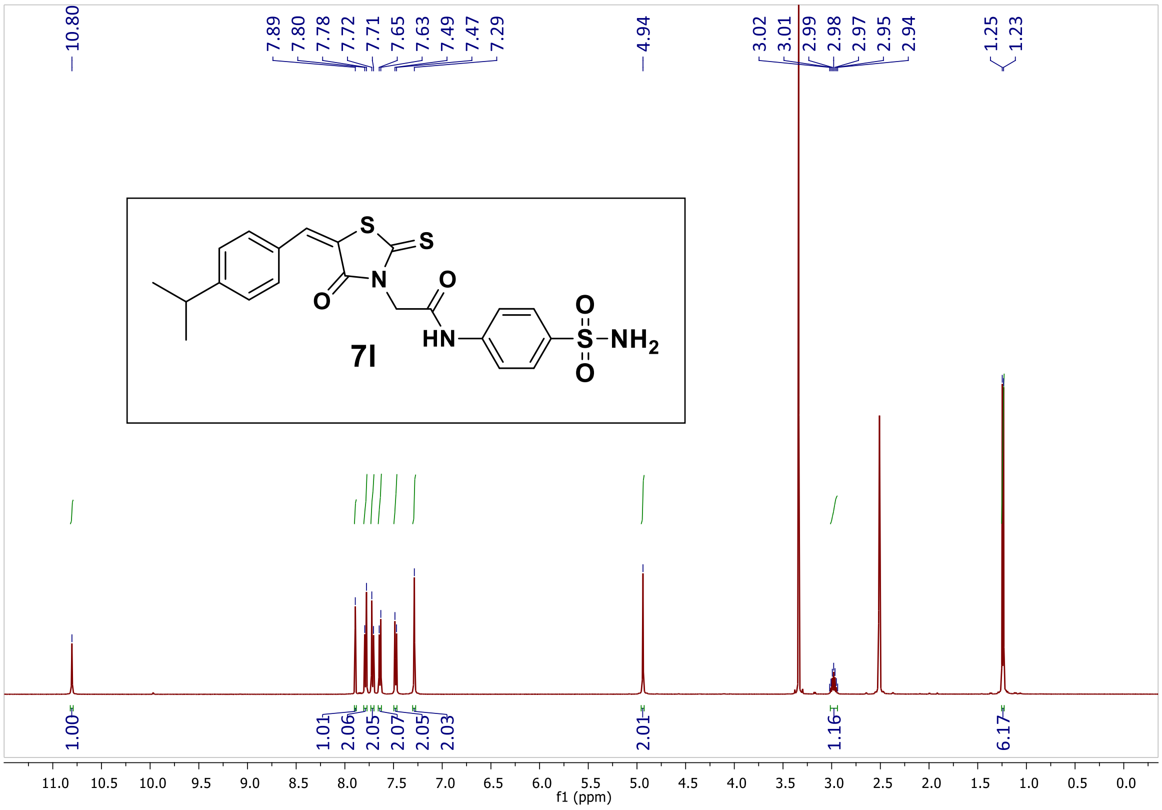


Figure S27. $^{1}$H NMR of **7l** in DMSO-$d_6$

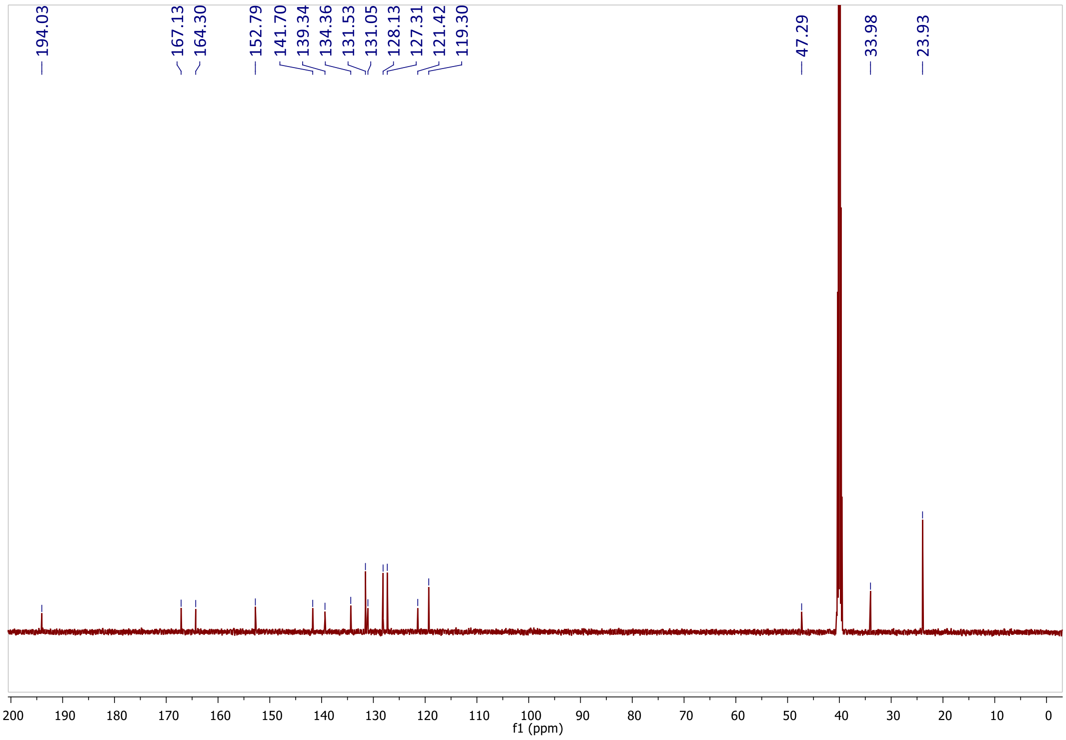


Figure S28. $^{13}$C NMR of **7l** in DMSO-$d_6$

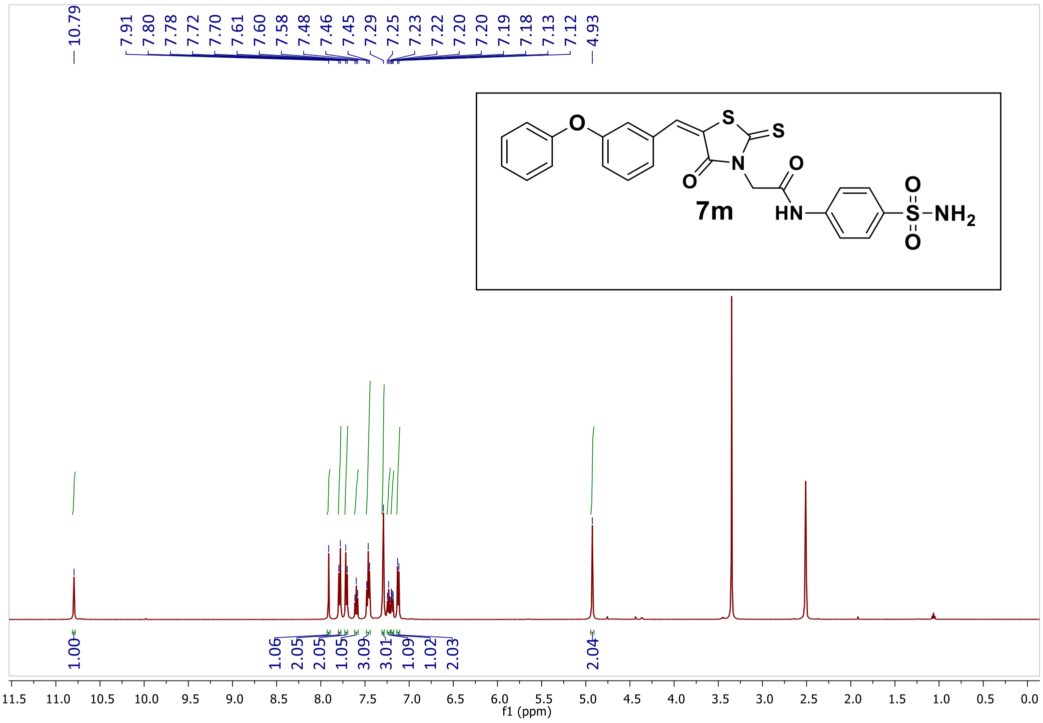


Figure S29. $^{1}$H NMR of **7m** in DMSO-$d_6$

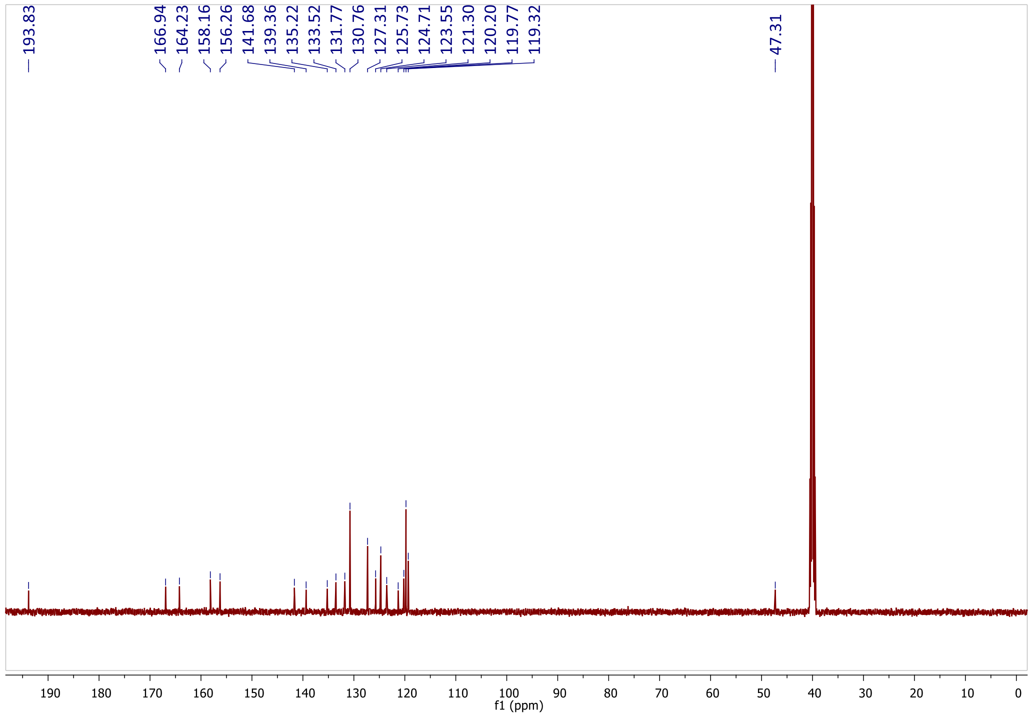


Figure S30. $^{13}$C NMR of **7m** in DMSO-$d_6$

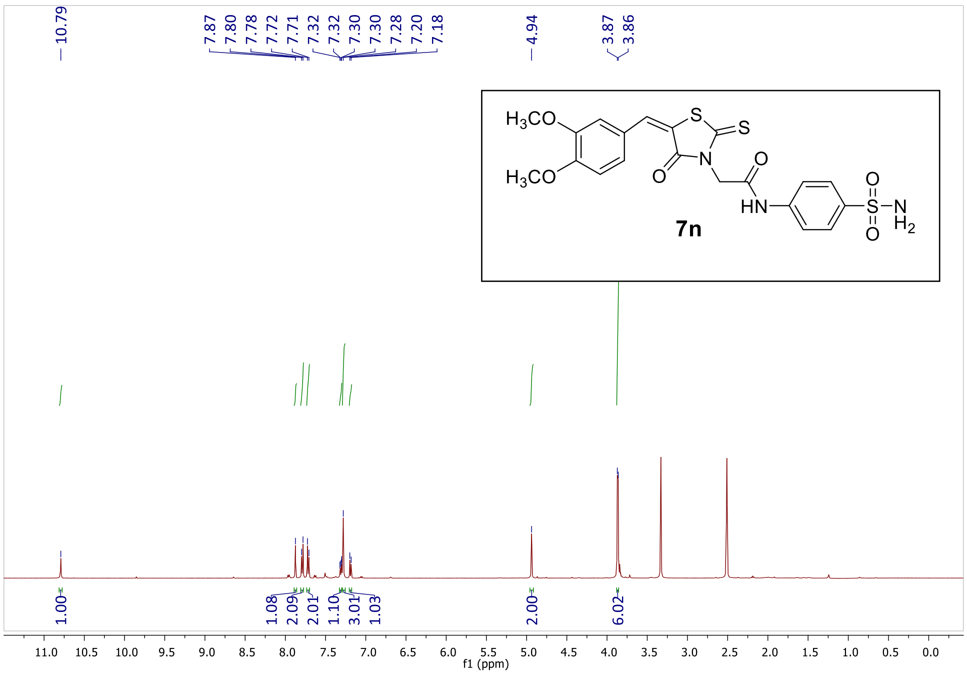

Figure S31. $^{1}H$ NMR of **7n** in DMSO-$d_6$

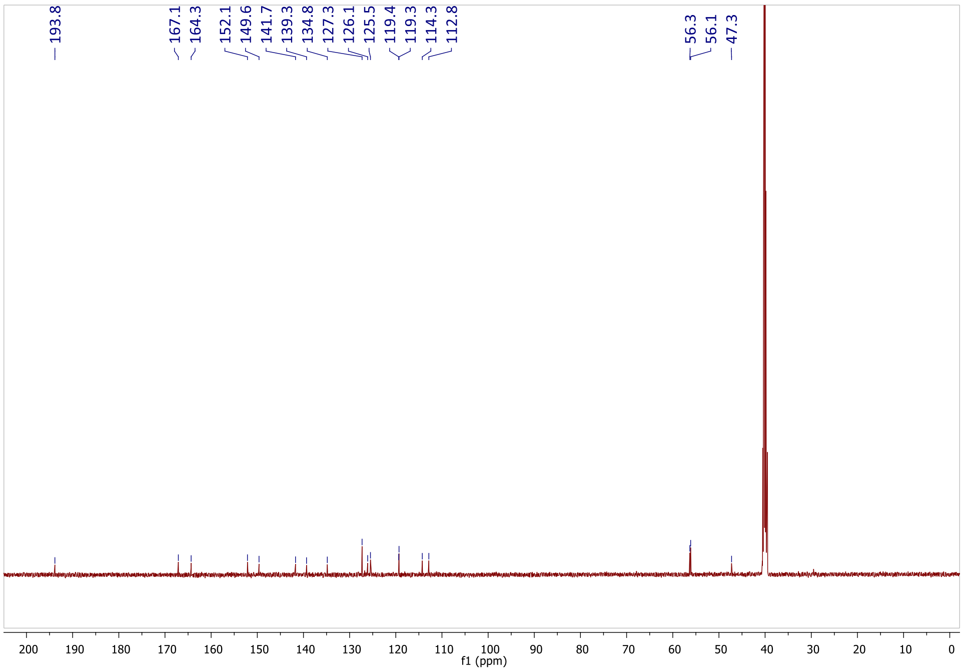

Figure S32. $^{13}C$ NMR of **7n** in DMSO-$d_6$

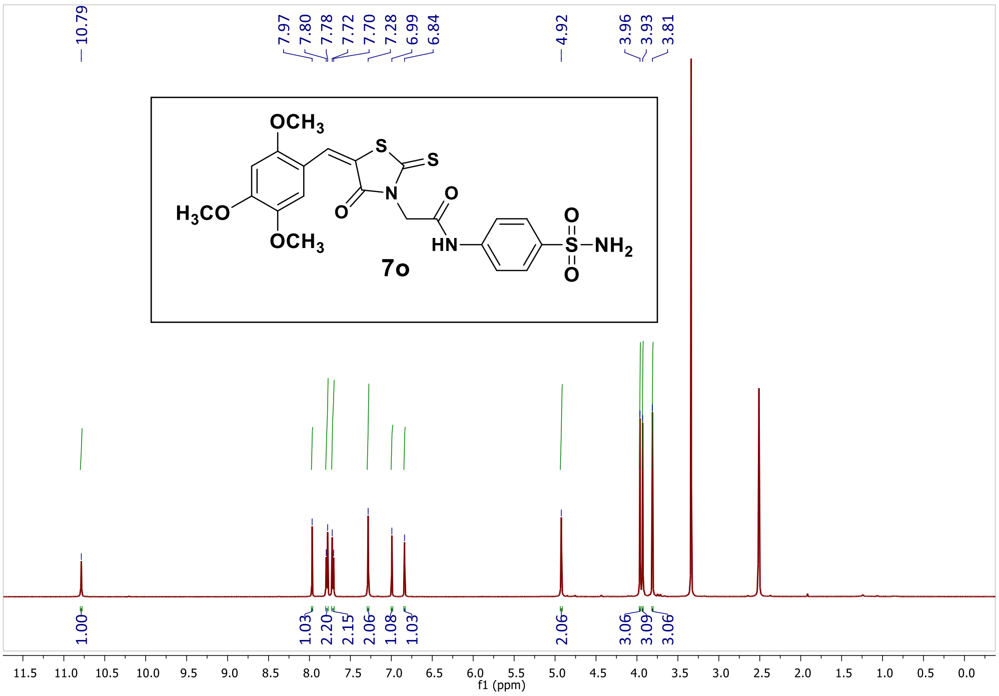


Figure S33. $^{1}H$ NMR of **7o** in DMSO-$d_6$

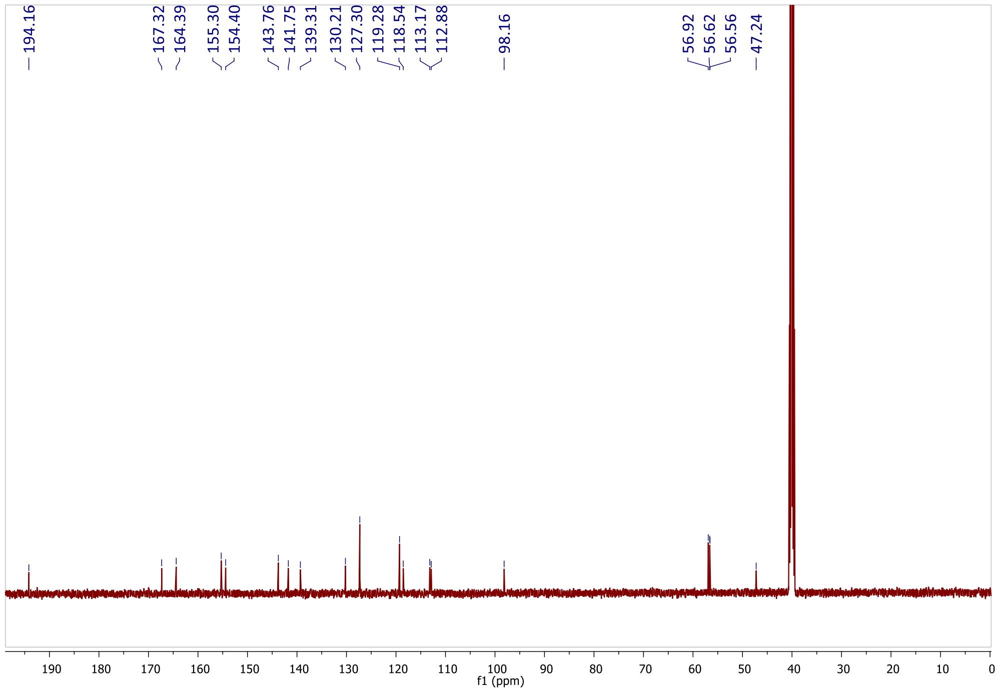


Figure S34. $^{13}C$ NMR of **7o** in DMSO-$d_6$

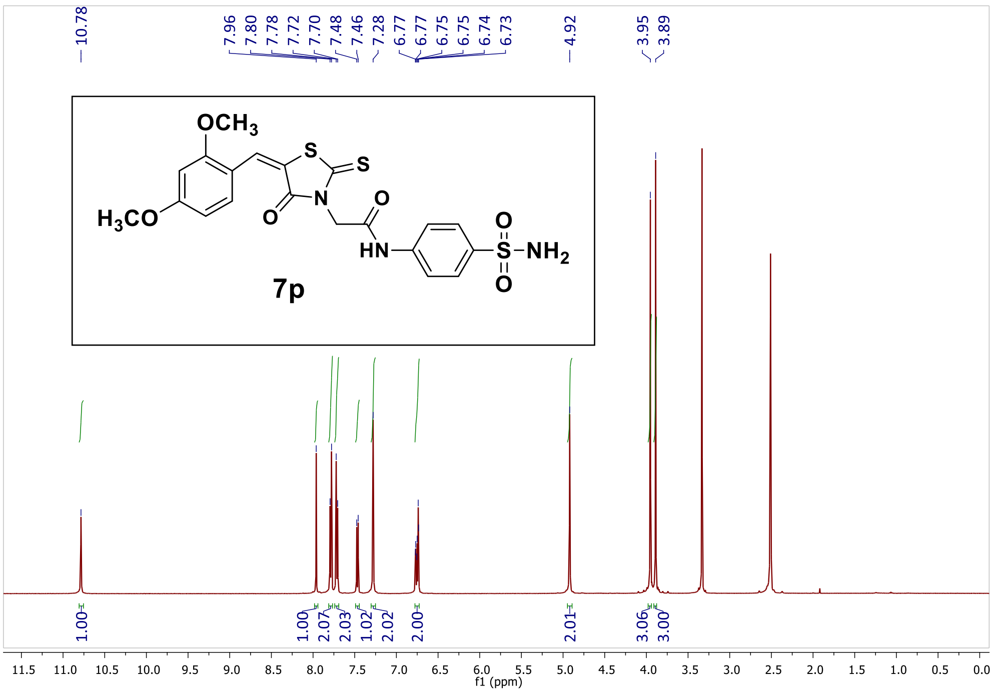


Figure S35. $^{1}$H NMR of **7p** in DMSO-$d_6$

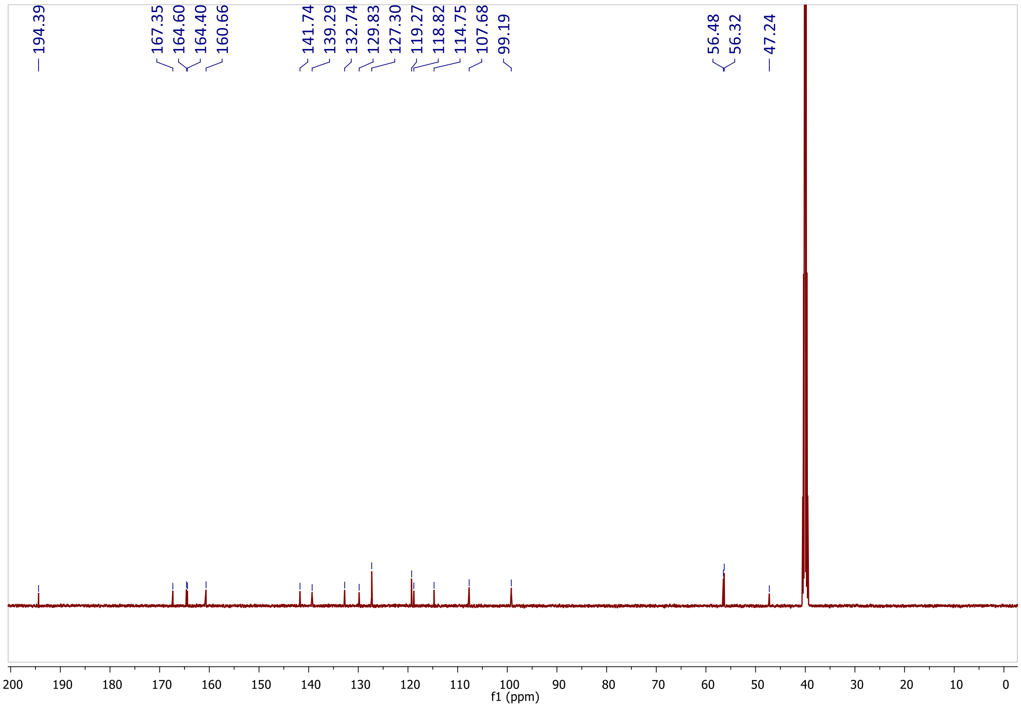


Figure S36. $^{13}$C NMR of **7p** in DMSO-$d_6$

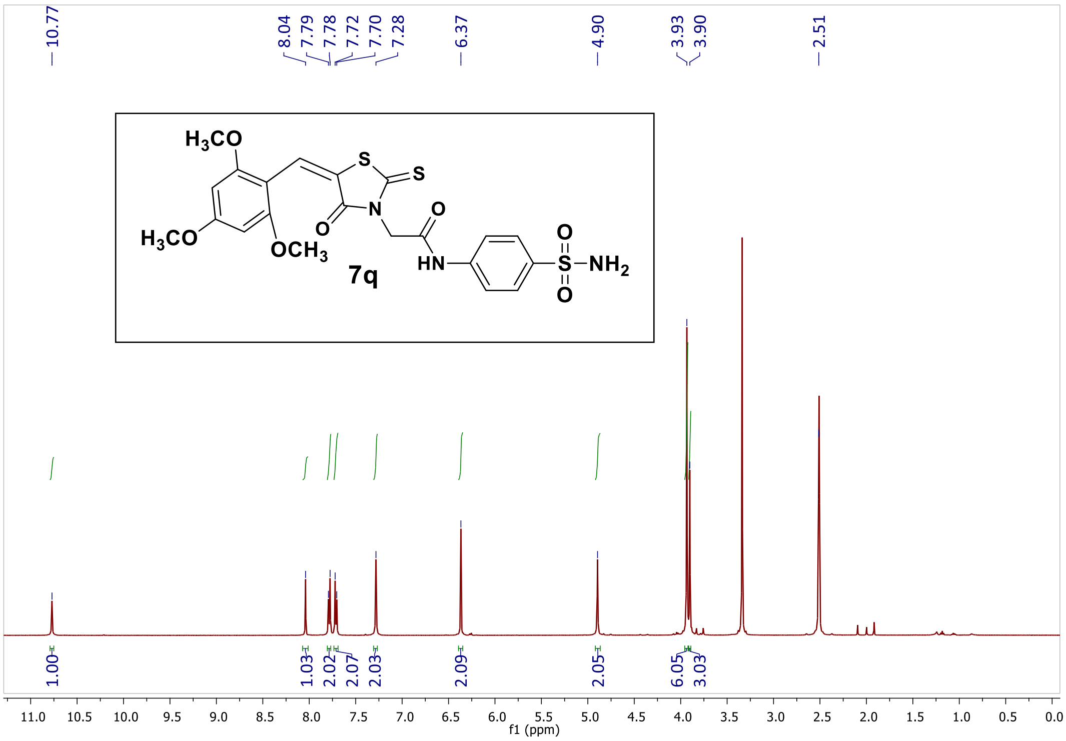


Figure S37. $^{1}$H NMR of **7q** in DMSO-*d*6

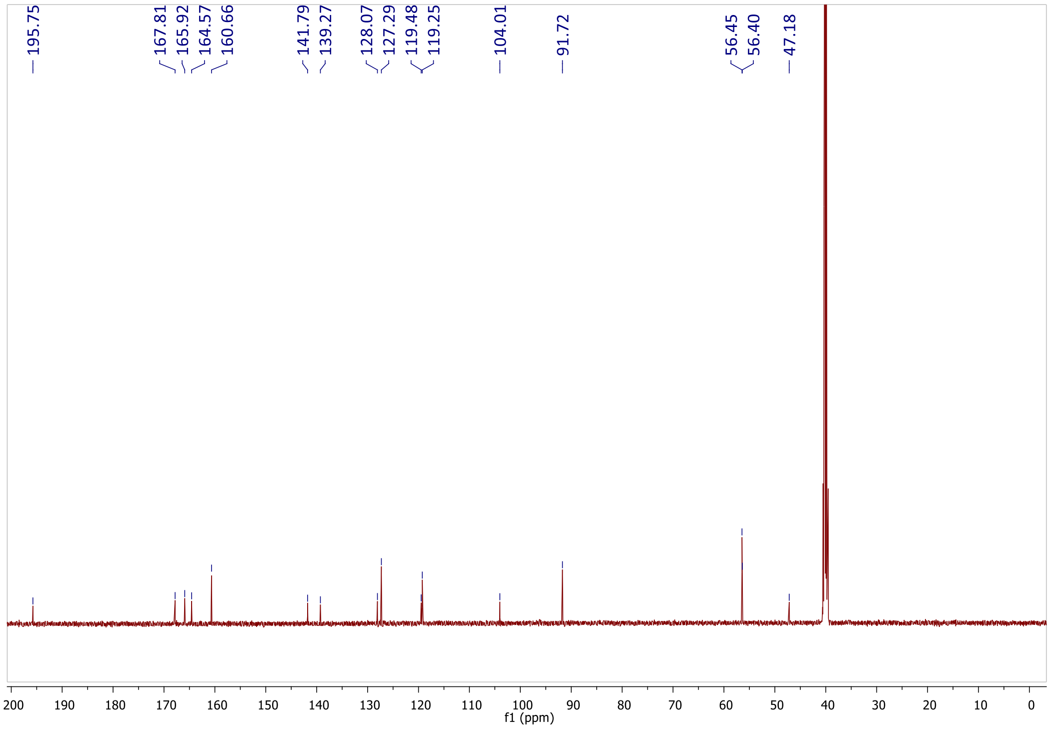


Figure S38. $^{13}$C NMR of **7q** in DMSO-*d*6

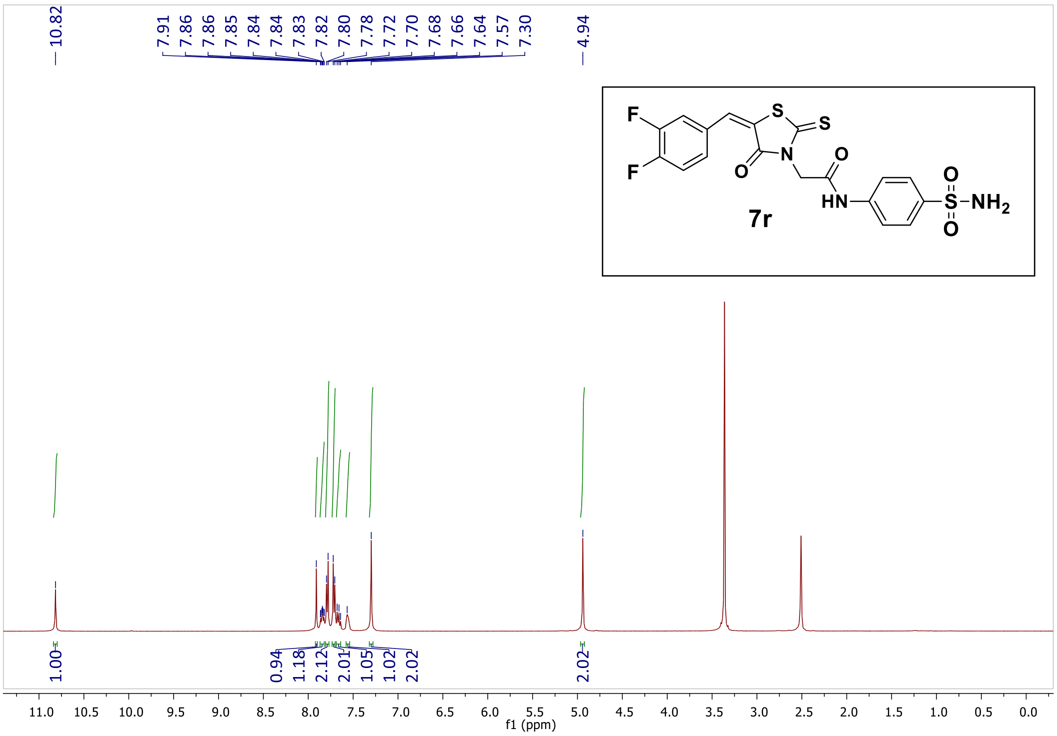


Figure S39. $^{1}$H NMR of **7r** in DMSO-$d_6$

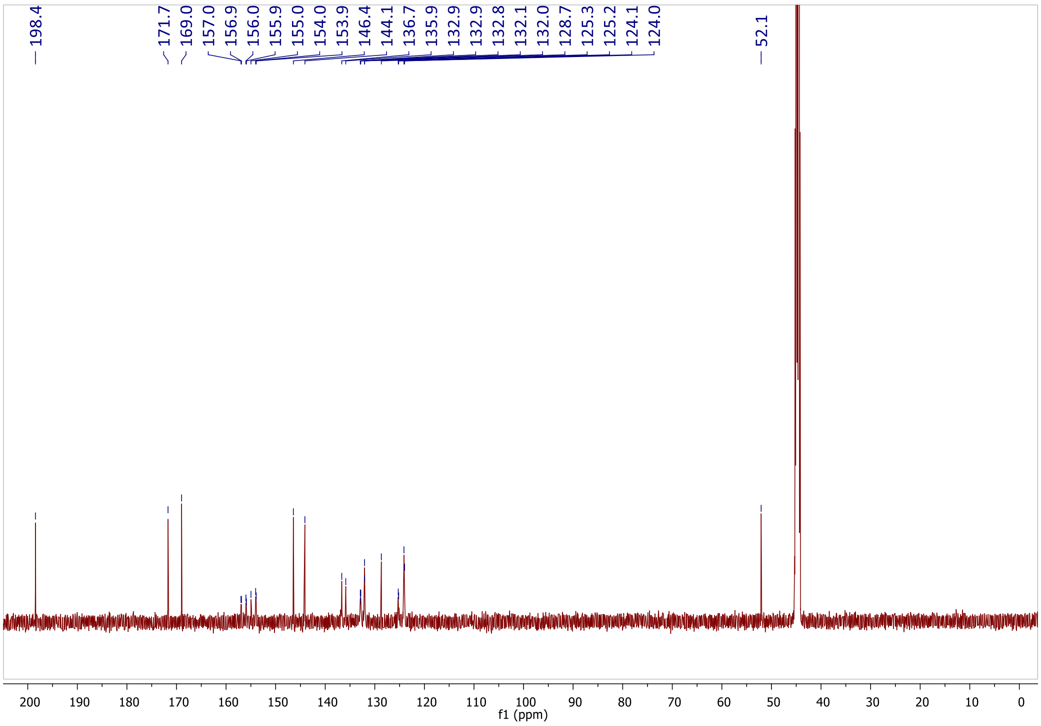


Figure S40. $^{13}$C NMR of **7r** in DMSO-$d_6$

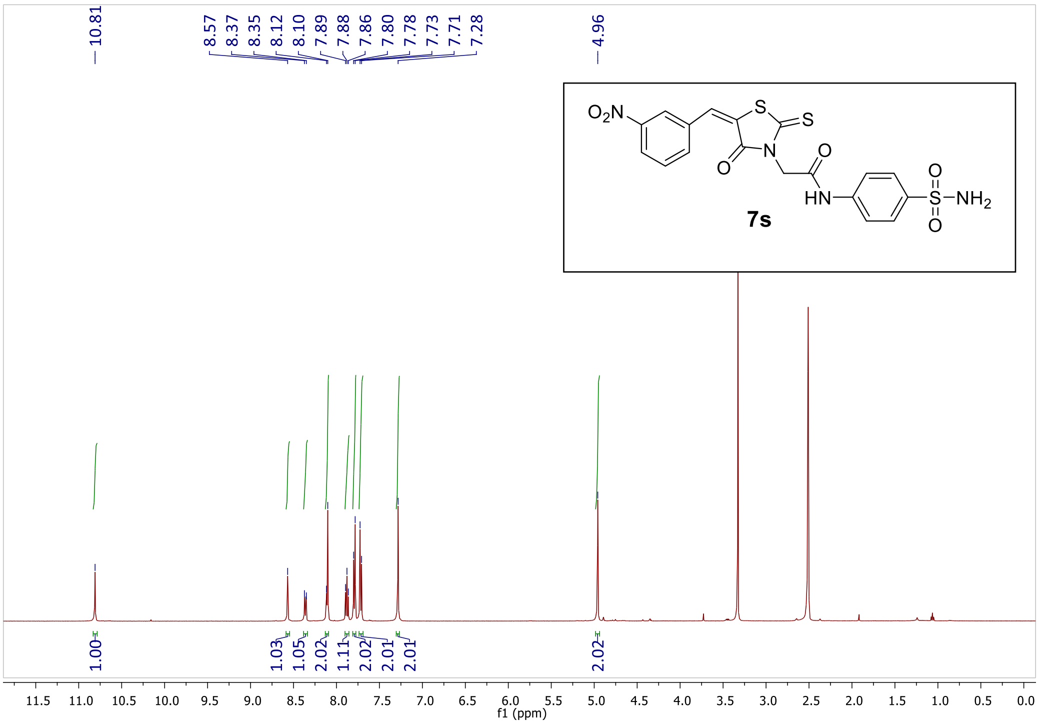


Figure S41. $^{1}$H NMR of **7s** in DMSO-$d_6$

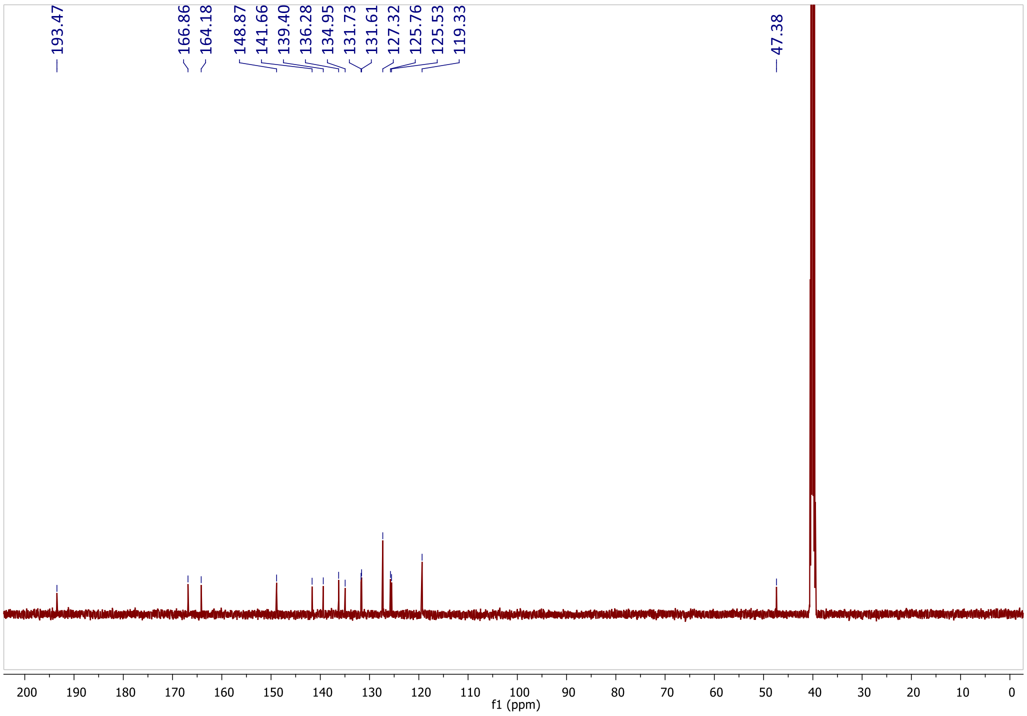


Figure S42. $^{13}$C NMR of **7s** in DMSO-$d_6$

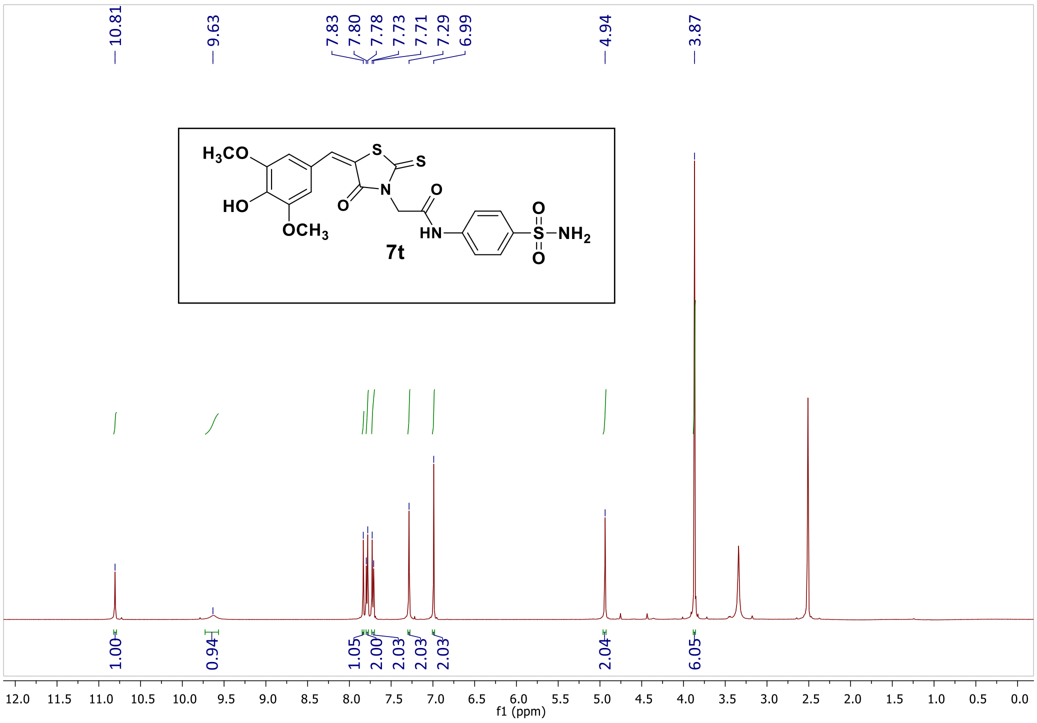


Figure S43. $^{1}$H NMR of **7t** in DMSO-$d_6$

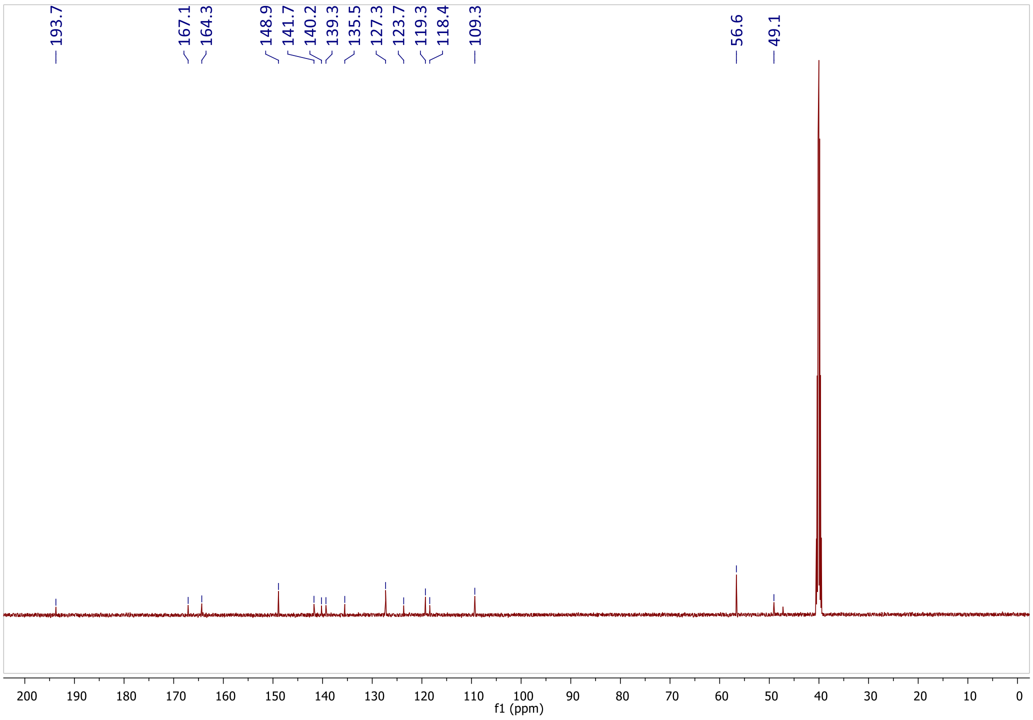


Figure S44. $^{13}$C NMR of **7t** in DMSO-$d_6$

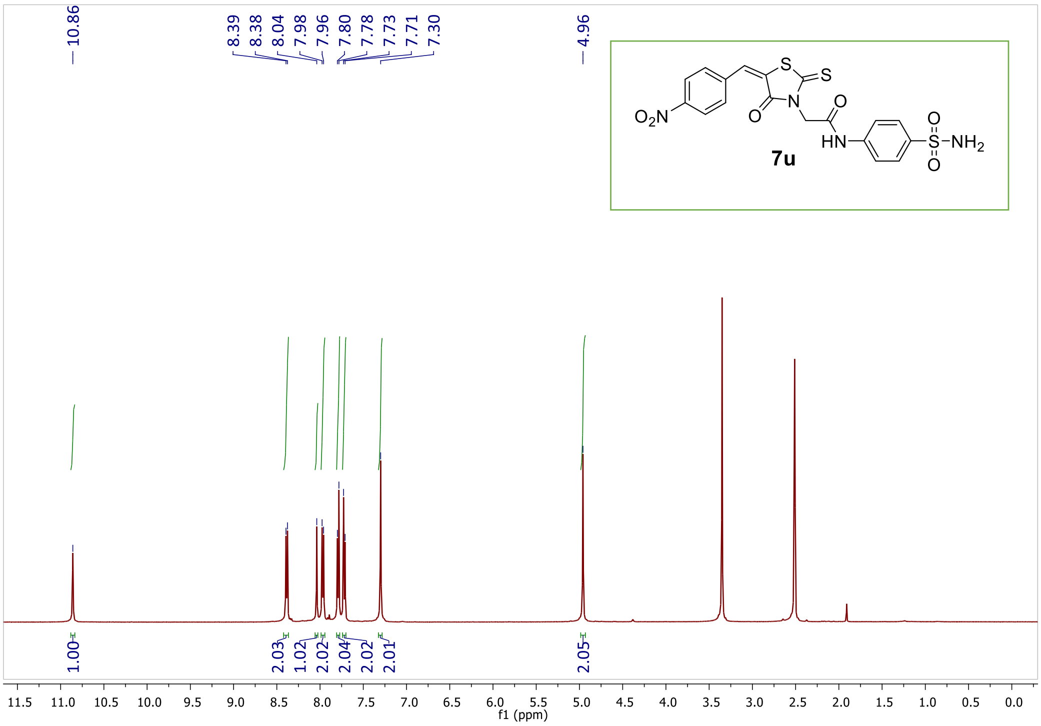


Figure S45. $^{1}$H NMR of **7u** in DMSO-$d_6$

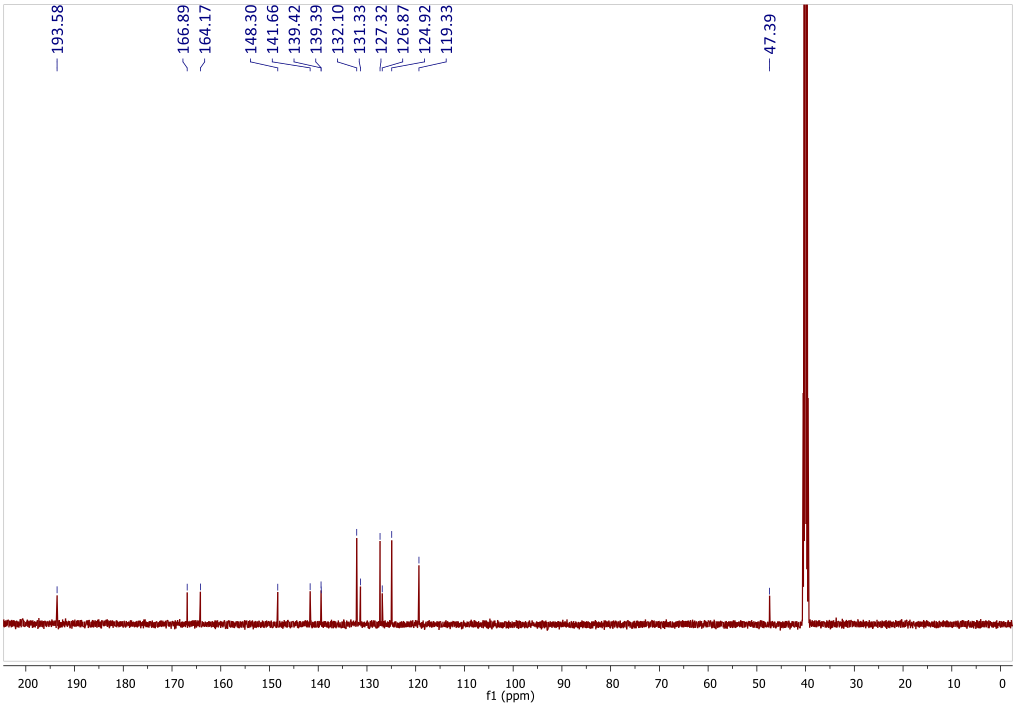


Figure S46. $^{13}$C NMR of **7u** in DMSO-$d_6$

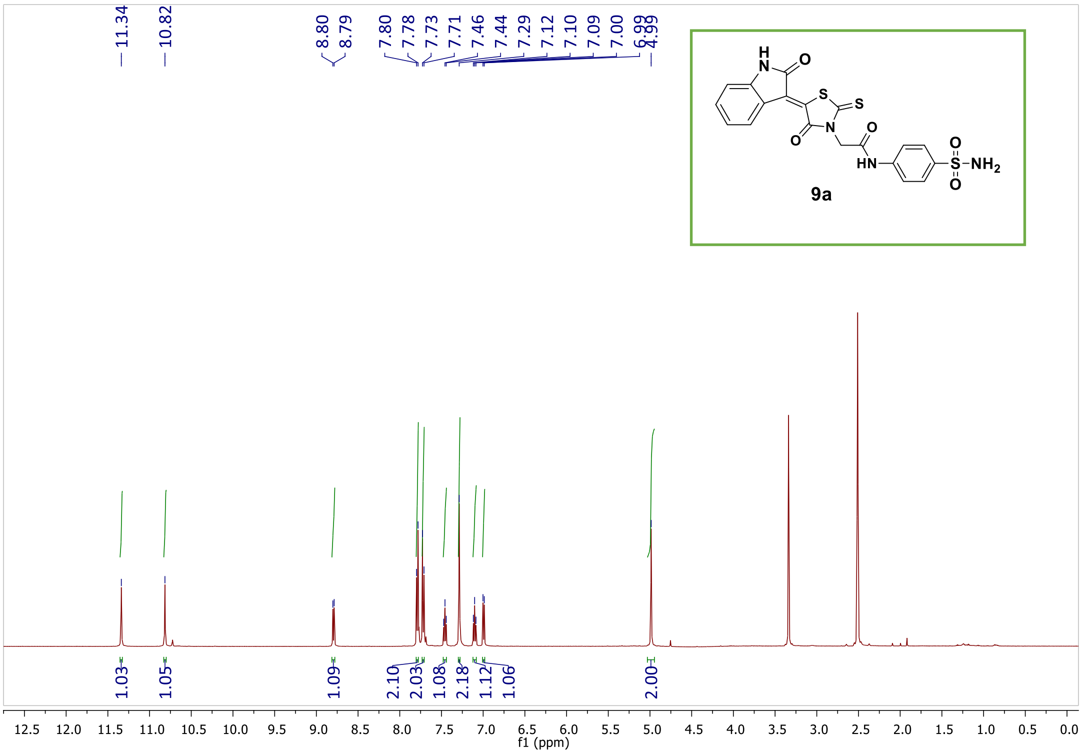


Figure S47. $^{1}$H NMR of **9a** in DMSO-$d_6$

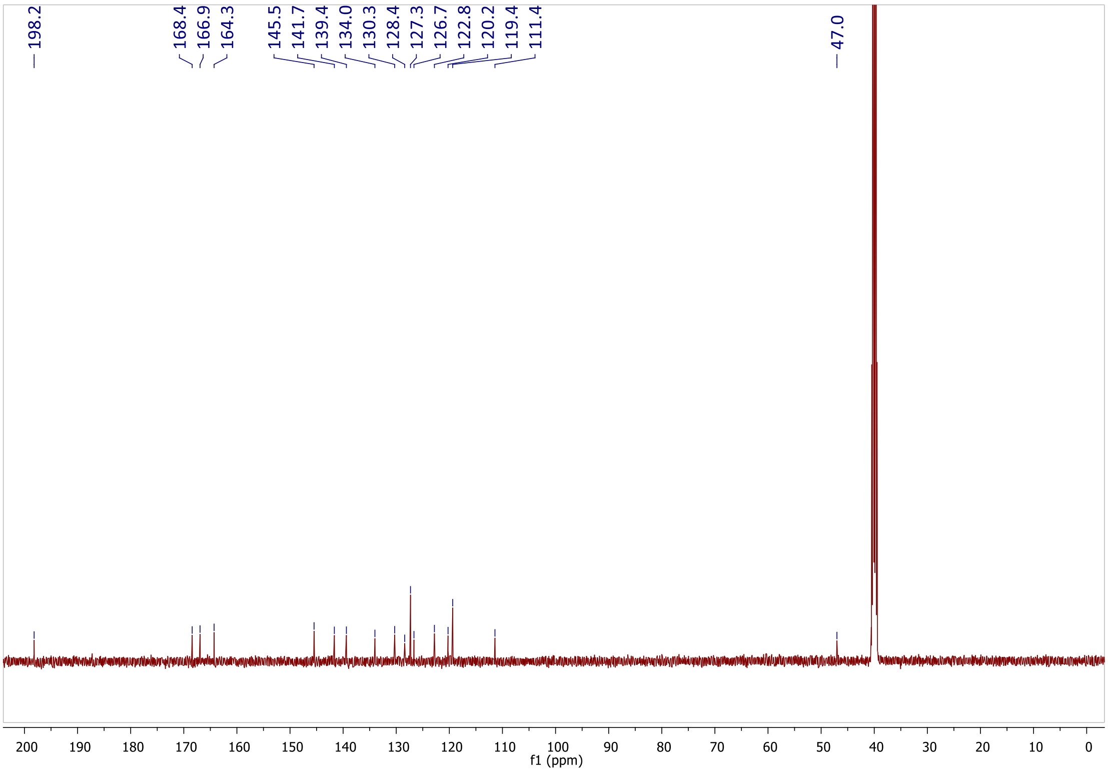


Figure S48. $^{13}$C NMR of **9a** in DMSO-$d_6$

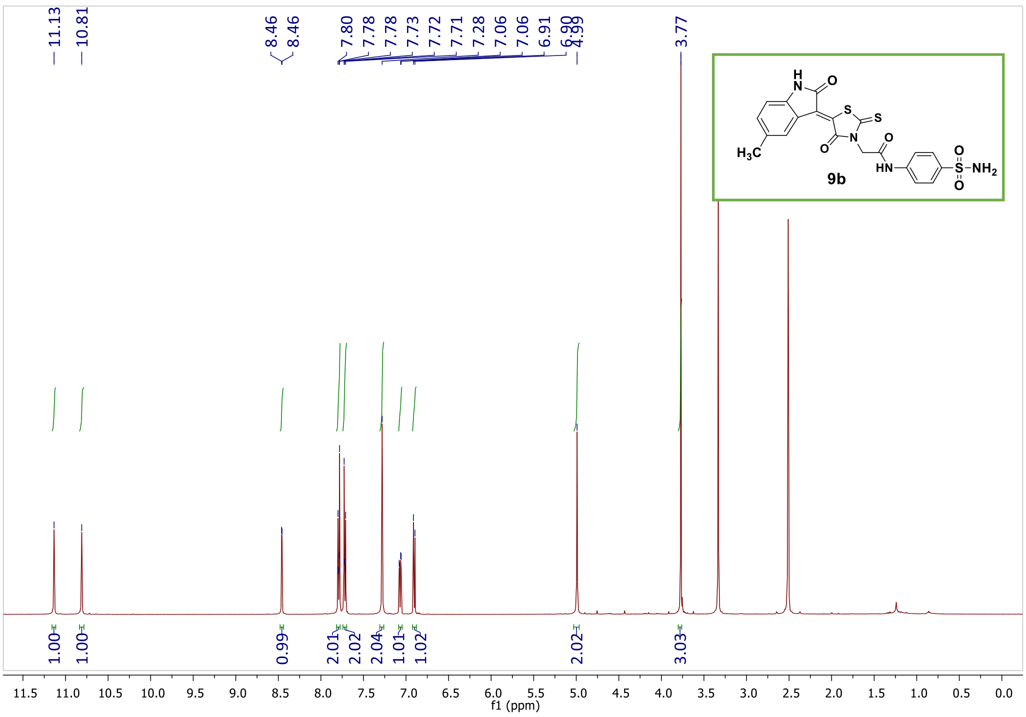


Figure S49. $^1$H NMR of **9b** in DMSO-$d_6$

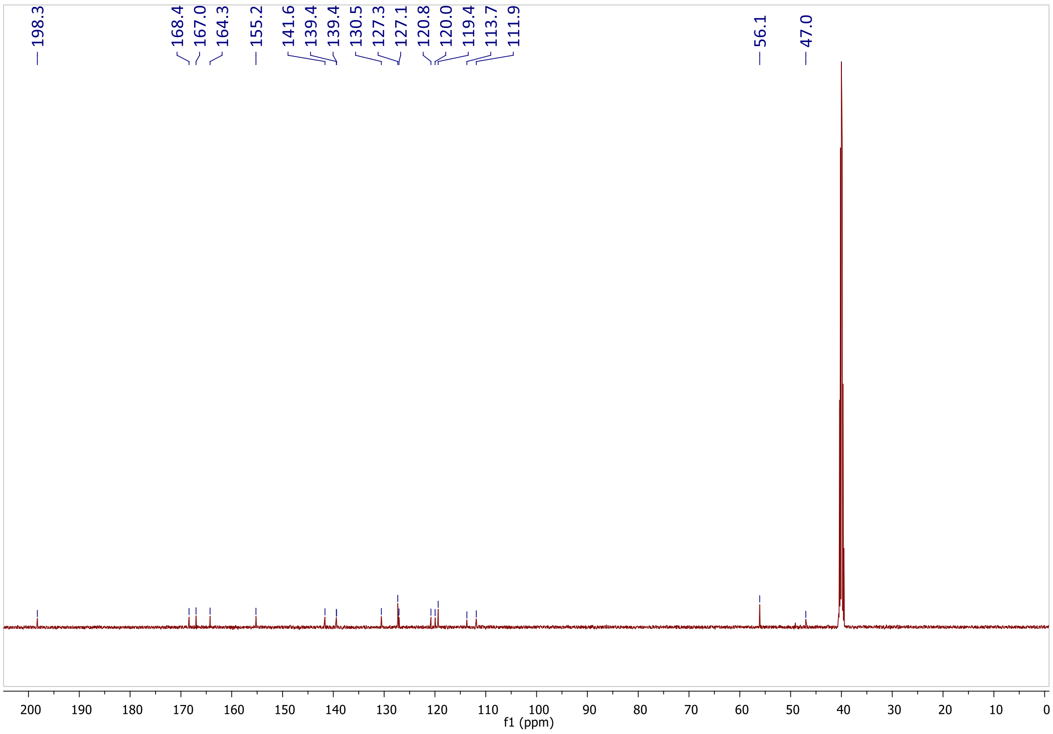


Figure S50. $^{13}$C NMR of **9b** in DMSO-$d_6$

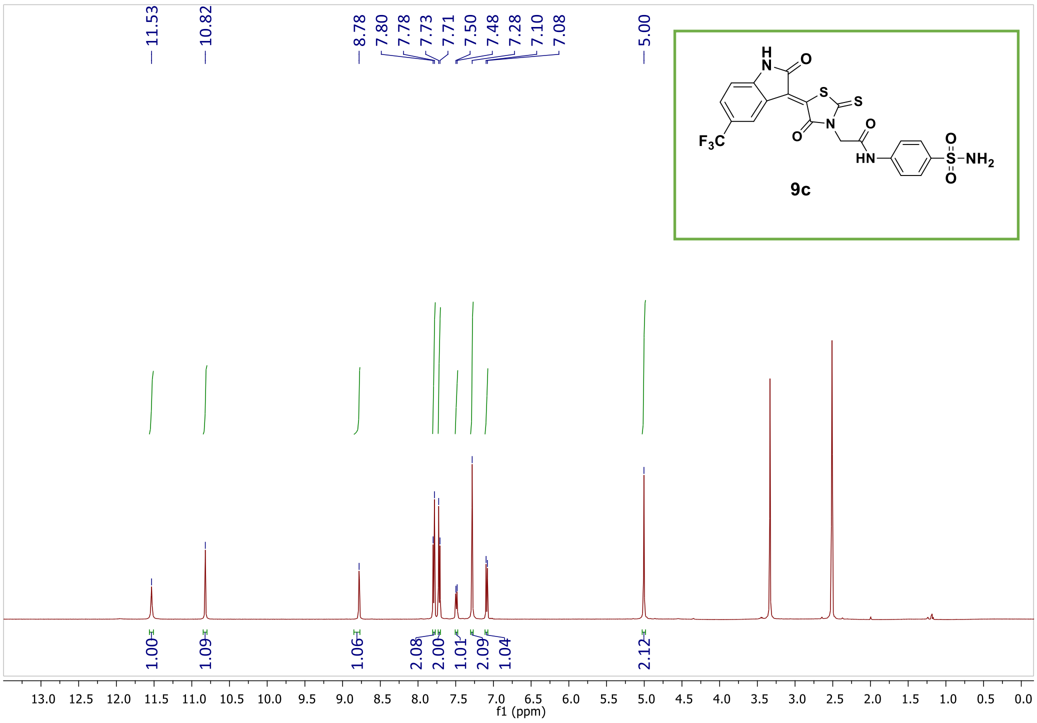


Figure S51. $^{1}$H NMR of **9c** in DMSO-$d_6$

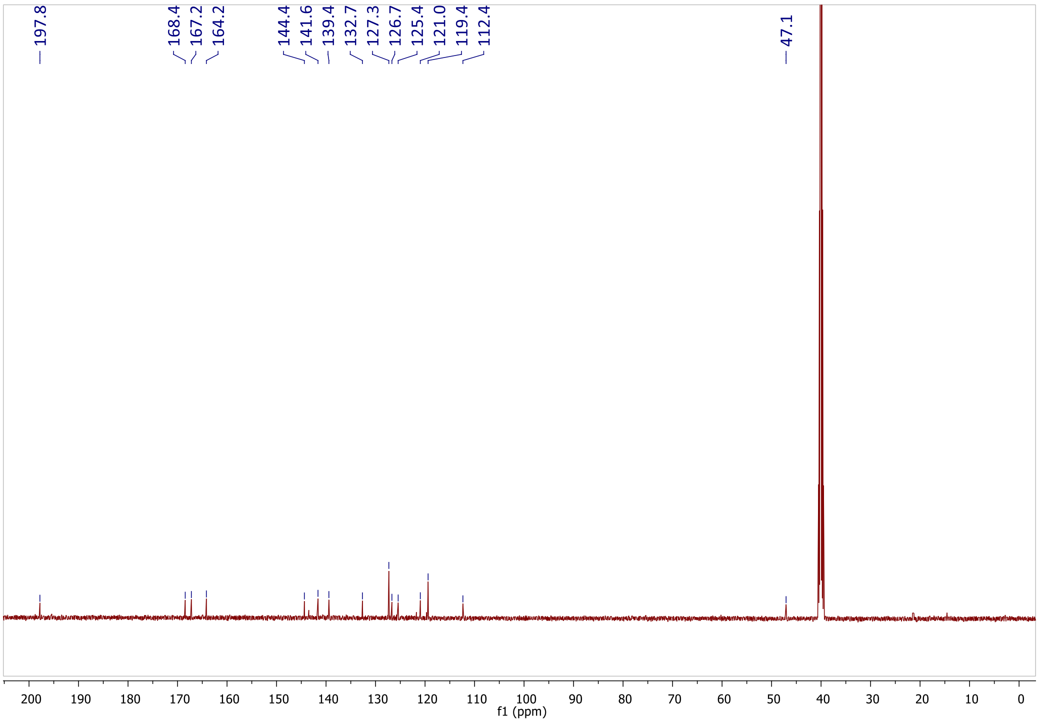


Figure S52. $^{13}$C NMR of **9c** in DMSO-$d_6$

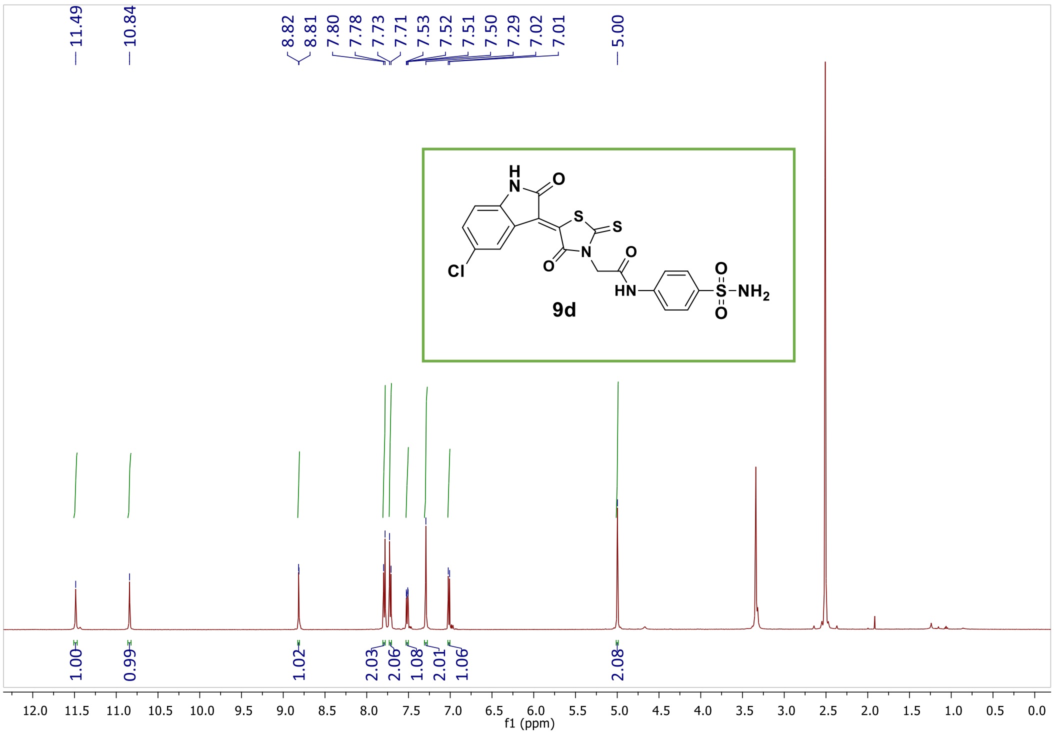


Figure S53. $^{1}$H NMR of **9d** in DMSO-$d_6$

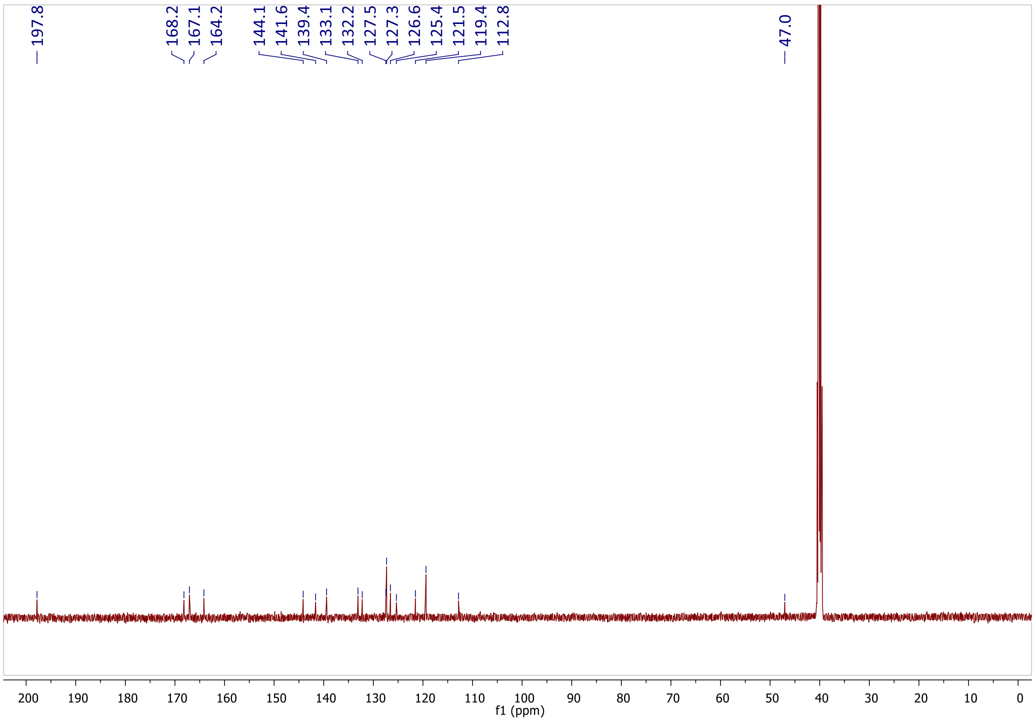


Figure S54. $^{13}$C NMR of **9d** in DMSO-$d_6$
